# Global, regional, and national years lived with disability due to blindness and vision loss from 1990 to 2019: Findings from the Global Burden of Disease Study 2019

**DOI:** 10.3389/fpubh.2022.1033495

**Published:** 2022-10-28

**Authors:** Shasha Li, Enlin Ye, Jiasheng Huang, Jia Wang, Yumei Zhao, Dongdong Niu, Suru Yue, Xueying Huang, Jie Liu, Xuefei Hou, Jiayuan Wu

**Affiliations:** ^1^Clinical Research Service Center, Affiliated Hospital of Guangdong Medical University, Zhanjiang, China; ^2^Guangdong Engineering Research Center of Collaborative Innovation Technology of Clinical Medical Big Data Cloud Service in Medical Consortium of West Guangdong Province, Affiliated Hospital of Guangdong Medical University, Zhanjiang, China

**Keywords:** years lived with disability, secular trend, Global Burden of Disease, blindness, vision loss

## Abstract

**Purpose:**

This study aimed to provide a comprehensive assessment of burden estimates and the secular trend of blindness and vision loss, as measured by years lived with disability (YLDs), at the global, regional, and national levels.

**Methods:**

The age-standardized YLD rates (ASYRs) due to blindness and vision loss and its subtypes, including moderate vision loss, severe vision loss, blindness, and presbyopia, from 1990 to 2019 were extracted from the Global Burden of Disease Study 2019 database. The estimated annual percentage changes (EAPCs) were calculated to quantify the temporal trends in the ASYRs due to blindness and vision loss.

**Results:**

In 2019, the global ASYRs per 100,000 population was 327.98 for blindness and vision loss, specifically, 85.81 for moderate vision loss, 74.86 for severe vision loss, 95.03 for blindness, and 62.27 for presbyopia. From 1990 to 2019, the ASYRs due to blindness and vision loss slightly decreased. Females showed higher ASYRs than males in 2019. The global highest ASYRs were observed in South Asia and sub-Saharan Africa. Negative associations were found between the burden estimates of blindness and vision loss and the sociodemographic index levels. The EAPCs of ASYRs in blindness and vision loss were significantly negatively correlated with the ASYRs in 1990 and positively correlated with human development indices in 2019.

**Conclusions:**

Globally, blindness and vision loss continue to cause great losses of healthy life. Reasonable resource allocation and health-service planning are needed for the prevention and early intervention of disabilities caused by vision loss.

## Introduction

Blindness and vision loss are enormous public-health challenges globally, which are associated with reduced economic, educational, and employment opportunities (1–4), as well as increased risk of death (5). In the elderly, blindness and vision loss can seriously reduce the quality of life (6) and increase the risk of cognitive impairment (7), and falls (8). In 2020, 295 million people suffered from moderate and severe vision impairment, 43.3 million people had blindness, and 510 million people had vision impairment due to uncorrected presbyopia (9). Furthermore, the Vision Loss Expert Group used years lived with disability (YLDs) to describe the burden of vision loss (9). In 2019, 22.6 million YLDs were caused by blindness and vision loss worldwide, with an increase of 20.3% since 2010. In terms of all causes of YLDs, blindness and low vision rank eighth in people aged 50–69 years and fourth in people aged over 70 years. This finding indicates that numerous individuals with visual impairment live for many years due to chronic disability. Indeed, this condition is a challenge to increase health-management services for the elderly particularly in aging societies.

The main causes of global blindness and vision loss are cataract, uncorrected refractive error, age-related macular degeneration, glaucoma, and diabetic retinopathy (9). The aging process is generally considered to be the most important factor affecting presbyopia, and the trend of aging of the world population has also greatly increased the number of presbyopia cases (10). In fact, cataracts and uncorrected refractive errors can be effectively treated with surgery and glasses, respectively (11). Implementing currently known effective treatments for avoidable vision loss can bring significant productivity gains to the global economy. However, eye-care services fail to keep up with the population growth and aging to meet people's needs, even in high-income areas, the problem of avoidable vision loss is not adequately addressed.

Previous studies reporting the burden of blindness and vision loss have focused on prevalence and incidence. These metrics are vital, but they provide limited assessment when reviewed individually. Their prevalence and incidence metrics assess injuries only on the basis of frequency and cannot easily reflect the degree and duration of disability (12). Years lived with disability (YLDs) express specified severity and duration. A previous article roughly describes the YLDs of vision loss, but no detailed systematic study on the trends of blindness and vision loss and its subtypes have been reported (9). Given that disability is a growing part of disease burden and health expenditures (13), understanding the latest information on YLD trends in blindness and vision loss and how they vary across countries is essential for planning appropriate health-system responses. In the present work, we used the study estimates from the Global Burden of Disease (GBD) 2019 to systematically analyze the burden, as measured by YLDs, due to blindness and vision loss and its subtypes (including moderate vision loss, severe vision loss, blindness, and presbyopia) at the global, regional, and national levels. We also determined how the burden due to blindness and vision loss is related to the socioeconomic development level as measured by human development indices (HDIs).

## Methods

### Study data

We extracted the age-standardized YLD rates (ASYRs) of blindness and vision loss and its subtypes at the global, regional, and national levels from 1990 to 2019 based on the GBD 2019 database. The general methods used in the GBD 2019 are described in detail on the official website (http://www.healthdata.org/gbd/). We obtained the sociodemographic index (SDI) within 1990 to 2019 from the GBD 2019 official website. Based on the SDI, 204 countries or territories were divided into five levels: low-, low–middle-, middle-, high–middle-, and high-SDI regions. SDI is a comprehensive indicator of the development level of each country based on lag-distributed income per capita, mean education for those 15 years old and older, and total fertility rate for those under 25 years old (14). The world was also geographically divided into 21 regions to observe the geographic disparities. The HDIs of countries were collected from the World Bank. We also compared the ASYRs due to blindness and vision loss and its subtypes in different age groups. The age stratification in the GBD 2019 were as follows: 5-year age group from age 0 to 95 and then a single category for >95 years old.

### Definitions

We used WHO criteria to classify the severity of vision loss according to vision in the better-seeing eye. The categories were moderate vision loss (defined as visual acuity of ≥6/60 and < 6/18), severe vision loss (visual acuity of ≥3/60 and < 6/60), blindness (visual acuity of < 3/60 or < 10% visual field around central fixation, although the visual-field definition is rarely utilized in population-based eye surveys), and presbyopia (near visual acuity of < 6/12 distance equivalent). YLDs represent the non-fatal component of burden and incorporate both the prevalence of a disease and the effect of the disease in terms of disability. YLDs for blindness and vision loss were calculated by the prevalence multiplied by disability weights (DWs) for the health state associated with blindness and vision loss. The basis of the DWs assessments is lay descriptions of health states, highlighting major functional consequences and symptom associated with each health state. DWs represents the magnitude of health loss associated with the vision loss, measured on a scale from 0 to 1, where 0 represents the equivalent of full health and 1 represents death (13). The DWs developed to describe the severity of different degrees of vision loss and the related functional loss were as follows: (1) moderate vision loss (DWs: 0.031, 95% CI: 0.019–0.049); (2) severe vision loss (DWs: 0.184, 95% CI: 0.125–0.258); and (3) blindness (DWs: 0.187, 95% CI: 0.124–0.0.260); (4) presbyopia (DWs: 0.011, 95% CI: 0.005–0.02).

### Statistical analysis

The goal of data standardization is to maximize data comparability. The ASYR corresponds to the YLDs per 100,000 population. The age-standardized rate (ASR) was calculated by summing up the products of the age-specific rates (*a*_*i*_, where *i* is the *i*th age class) and number of persons (or the weight; *w*_*i*_) in the same age subgroup *i* of the selected reference standard population and then dividing the sum of the standard population weights (15). The age-standardized populations in the GBD were calculated using the GBD world-population age standard.


$$\begin{array}{l} ASR=\;\frac{\sum_{i=1}^{A}a_{i}w_{i}}{\sum_{i=1}^{A}w_{i}}\;\times 100,000 \end{array}$$

The estimated annual percentage change (EAPC) is commonly used to reflect the variation tendency of ASRs over a specified interval. The natural logarithm of the regression-line fit to ASR is *y* = *a* + *bx* + *e*, where *x* = the calendar year. EAPC is calculated as 100 × [exp(*b*) – 1], and its 95% uncertainty interval (UI) can also be obtained from a linear-regression model (16). If the estimated EAPCs and the lower bound of its 95% UI are both >0, then ASRs would exhibit an increasing trend; if the estimated EAPC and the upper bound of its 95% UI are < 0, then ASRs would trend downwards; otherwise, ASRs would be considered stable.

All analyses were conducted using R program (version 4.0.5, R Core Team). *P*-values < 0.05 was considered statistically significant.

## Results

### Burden of blindness and vision loss at the global level

**Figure 1:**
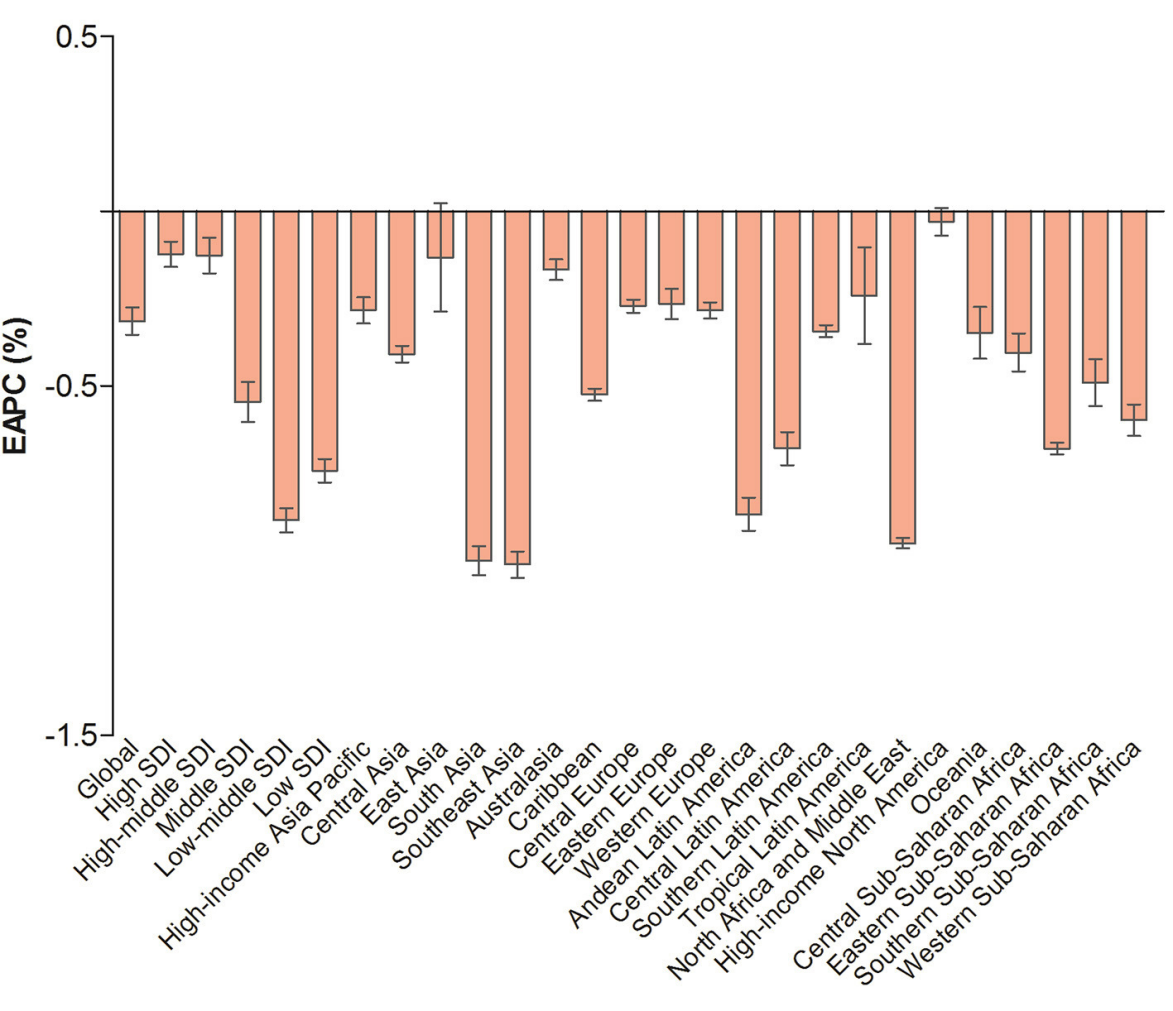
The EAPC of age-standardized YLD rates due to blindness and vision loss at the global and regional levels. EAPC, estimated annual percentage change; YLD, years lived with disability; SDI, human development index.

Globally, the ASYRs due to blindness and vision loss decreased from 359.46 (95% UI: 247.58 to 500.65) in 1990 to 327.98 (95% UI: 222.49 to 465.64) in 2019 per 100,000 population by an EAPC of −0.32 (95% UI: −0.35 to −0.28; Figure 1 and Table 1). The global ASYRs per 100,000 population increased from 94.26 (95% UI: 56.5 to 149.31) in 1990 to 95.81 (95% UI: 57.5 to 151.45) in 2019 with the EAPC being 0.11 (95% UI: 0.08 to 0.14) for moderate vision loss, from 76.75 (95% UI: 51.38 to 110.53) in 1990 to 74.86 (95% UI: 49.97 to 107.67) in 2019 with the EAPC being 0.02 (95% UI: −0.05 to 0.09) for severe vision loss, from 129.69 (95% UI: 86.96 to 183.86) in 1990 to 95.03 (95% UI: 63.47 to 135.11) in 2019 at −1.10 per year (95% UI: −1.15 to −1.05) for blindness, and from 58.76 (95% UI: 26.80 to 117.68) in 1990 to 62.27 (95% UI: 28.12 to 124.55) in 2019 at 0.05 per year (95% UI: −0.01 to 0.11) for presbyopia (Supplementary Table 1 and Supplementary Figure 1).

**Table 1:** Age-standardized rates of years lived with disability due to blindness and vision loss in 2019 and their temporal trend from 1990 to 2019 at the global and regional levels.

	**ASYRs in 1990** **(per 10^5^ population, 95% UI)**	**ASYRs in 2019** **(per 10^5^ population, 95% UI)**	**EAPC (1990–2019, 95% UI)**
**Global**	359.46 (247.58–500.65)	327.98 (222.49–465.64)	−0.32 (−0.35 to −0.28)
**Sex**
Male	352.39 (242.97–492.25)	310.72 (211.09–441.46)	−0.44 (−0.48 to −0.41)
Female	366.69 (252.39–511.54)	343.51 (232.72–487.58)	−0.22 (−0.26 to −0.18)
**Subtypes**
Moderate vision loss	94.26 (56.50–149.31)	95.81 (57.50–151.45)	0.11 (0.08 to 0.14)
Severe vision loss	76.75 (51.38–110.53)	74.86 (49.97–107.67)	0.02 (−0.05 to 0.09)
Blindness	129.69 (86.96–183.86)	95.03 (63.47–135.11)	−1.10 (−1.15 to −1.05)
Presbyopia	58.76 (26.80–117.68)	62.27 (28.12–124.55)	0.05 (−0.01 to 0.11)
**Socio-demographic index**
High SDI	133.46 (91.45–186.76)	128.81 (87.55–180.54)	−0.12 (−0.16 to −0.09)
High-middle SDI	285.89 (192.85–408.23)	273.54 (183.45–395.81)	−0.13 (−0.18 to −0.08)
Middle SDI	456.03 (314.38–634.23)	386.76 (263.39–545.67)	−0.55 (−0.60 to −0.49)
Low-middle SDI	651.46 (452.03–903.23)	504.16 (345.21–711.47)	−0.88 (−0.92 to −0.85)
Low SDI	658.92 (457.35–911.44)	536.60 (368.64–748.69)	−0.74 (−0.78 to −0.71)
**Geographic region**
High-income Asia Pacific	125.13 (85.56–173.56)	115.49 (79.12–160.99)	−0.28 (−0.32 to −0.25)
Central Asia	349.41 (238.01–495.89)	313.14 (210.67–451.13)	−0.41 (−0.43 to −0.39)
East Asia	302.55 (204.56–436.49)	283.93 (190.99–415.34)	−0.13 (−0.29 to 0.02)
South Asia	796.23 (552.37–1,101.68)	601.96 (410.96–850.30)	−1.00 (−1.04 to −0.96)
Southeast Asia	607.30 (425.63–831.11)	459.51 (321.29–634.54)	−1.01 (−1.05 to −0.97)
Australasia	128.61 (87.34–181.28)	121.10 (81.70–170.50)	−0.17 (−0.20 to −0.14)
Caribbean	326.40 (225.92–454.60)	280.80 (191.68–397.46)	−0.53 (−0.54 to −0.51)
Central Europe	195.52 (128.29–292.09)	181.12 (117.93–272.07)	−0.27 (−0.29 to −0.25)
Eastern Europe	294.47 (196.74–427.05)	280.46 (184.03–418.02)	−0.27 (−0.31 to −0.22)
Western Europe	162.34 (112.18–224.98)	148.47 (101.61–206.88)	−0.28 (−0.31 to −0.26)
Andean Latin America	455.55 (318.92–631.02)	365.76 (251.74–515.57)	−0.87 (−0.92 to −0.82)
Central Latin America	413.33 (285.57–574.61)	337.46 (229.53–474.26)	−0.68 (−0.73 to −0.63)
Southern Latin America	186.40 (128.76–258.13)	167.07 (114.36–235.32)	−0.34 (−0.36 to −0.33)
Tropical Latin America	433.78 (304.21–598.07)	366.50 (253.40–507.67)	−0.24 (−0.38 to −0.10)
North Africa and Middle East	491.24 (342.57–669.87)	371.60 (257.70–515.21)	−0.95 (−0.97 to −0.94)
High-income North America	106.50 (72.41–149.11)	105.44 (71.92–147.97)	−0.03 (−0.07 to 0.01)
Oceania	431.35 (296.57–600.75)	389.66 (263.51–549.13)	−0.35 (−0.42 to −0.27)
Central Sub-Saharan Africa	340.58 (230.77–488.35)	299.53 (199.71–440.82)	−0.41 (−0.46 to −0.35)
Eastern Sub-Saharan Africa	569.48 (392.25–789.90)	472.04 (324.82–659.32)	−0.68 (−0.70 to −0.66)
Southern Sub-Saharan Africa	484.71 (329.63–695.77)	426.22 (283.82–639.01)	−0.49 (−0.56 to −0.42)
Western Sub-Saharan Africa	599.14 (416.87–822.68)	512.06 (351.94–718.37)	−0.60 (−0.64 to −0.55)

YLDs, years lived with disability; ASYR, age-standardized YLD rate; EAPC, estimated annual percentage change; UI, uncertainty interval.

Females had higher ASYRs than males in 2019 in terms of blindness and vision loss and its subtypes (Table 1 and Supplementary Table 1). The sex-specific ASYRs due to blindness and vision loss in 2019 decreased in both sexes compared with those in 1990. For females, ASYRs increased in moderate vision loss (EAPC = 0.15, 95% UI: 0.12 to 0.19), severe vision loss (EAPC = 0.11, 95% UI: 0.04 to 0.19), and presbyopia (EAPC = 0.13, 95% UI: 0.07 to 0.18) from 1990 to 2019. For males, ASYRs increased in moderate vision loss (EAPC = 0.06, 95% UI: 0.03 to 0.09) during the observed period (Supplementary Table 1).

In 2019, the highest ASYRs appeared in the low-middle and low-SDI regions, and the lowest ASYRs appeared in the high-SDI regions in terms of blindness and vision loss (Table 1) and its subtypes (Supplementary Table 1). An increasing trend was observed in the high, high-middle, and middle SDI regions for moderate vision loss, in the high-middle SDI region for severe vision loss, and in the high SDI region for presbyopia during the observed period (Figure 1 and Supplementary Figure 1).

**Figure 2:**
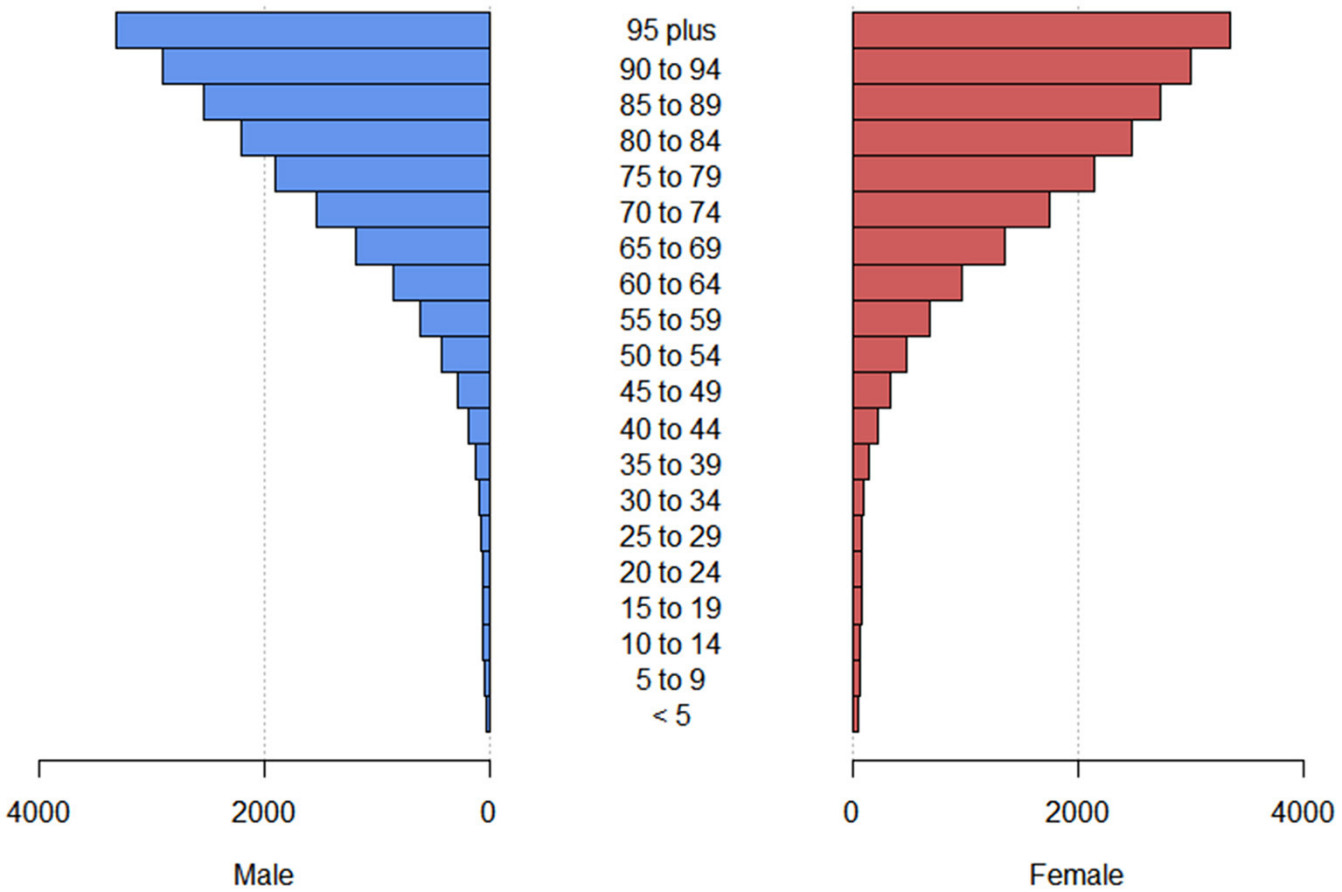
The crude YLD rate due to blindness and vision loss at different age groups globally. YLD, years lived with disability.

We also analyzed the ASYRs due to blindness and vision loss for males and females in different age groups. YLDs increased with age in terms of blindness and vision loss and its subtypes except for presbyopia, among which YLDs were highest in the age group of 80–84 years (Figure 2 and Supplementary Figure 2).

### Burden of blindness and vision loss at the regional level

In 2019, the ASYRs due to blindness and vision loss was highest in South Asia (601.96, 95% UI: 410.96 to 850.3, per 100,000 population) and lowest in high-income North America (105.44, 95% UI: 71.92 to 147.97, per 100,000 population). For the temporal trend of ASYRs, the most significant decrease was detected in Southeast Asia (EAPC = −1.01, 95% UI: −1.05 to −0.97), followed by South Asia and North Africa and Middle East (Table 1 and Figure 1).

In 2019, South Asia had the highest ASYRs due to moderate (166.77, 95% UI: 99.24 to 262.04, per 100,000 population) and severe (160.29, 95% UI: 106.66 to 230.74, per 100,000 population) vision loss. The highest ASYRs was observed in Western Sub-Saharan Africa for blindness (200.34, 95% UI: 134.64 to 286.93, per 100,000 population) and in Southern Sub-Saharan Africa for presbyopia (157.32, 95% UI: 69.25 to 311.32, per 100,000 population; Supplementary Table 1). East Asia had the largest increase in ASYRs due to moderate (EAPC = 0.42, 95% UI: 0.30 to 0.54) and severe (EAPC = 0.67, 95% UI: 0.33 to 1.01) vision loss, whereas Eastern Europe showed the greatest increase for presbyopia (EAPC = 0.09, 95% UI: 0.01 to 0.18) (Supplementary Fiugre 1).

### Burden of blindness and vision loss at the national level

**Figure 3:**
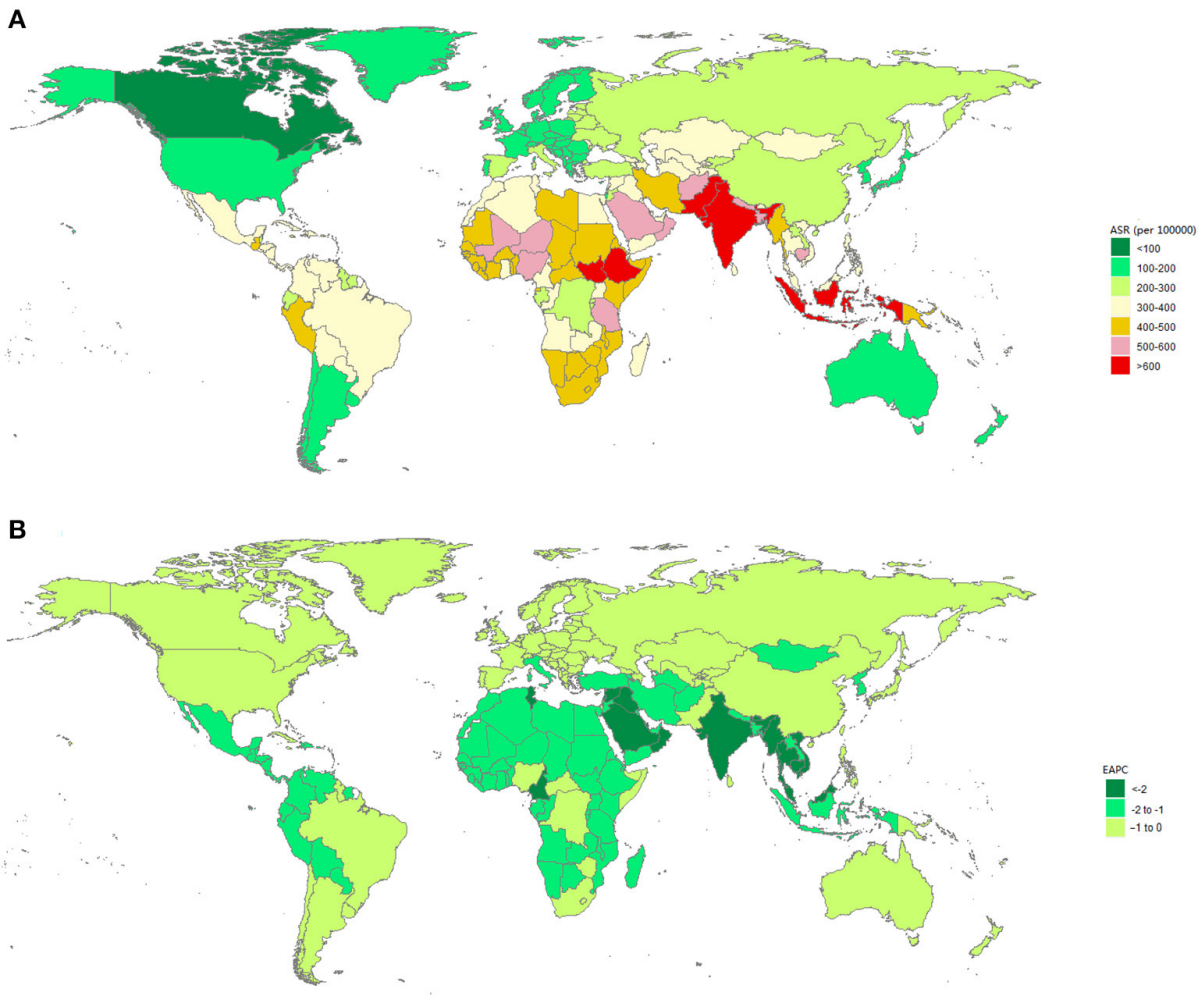
The global burden due to blindness and vision loss in 204 countries or territories. **(A)** The age-standardized YLD rate due to blindness and vision loss in 2019. **(B)** The EAPC of age-standardized YLD rate due to blindness and vision loss from 1990 to 2019. ASR, age-standardized rate; EAPC, estimated annual percentage change; YLD, years lived with disability.

In 2019, South Sudan (726.48, 95% UI: 506.55 to 1,003.08; per 100,000 population) had the highest ASYRs due to blindness and vision loss (Supplementary Table 2), followed by Pakistan and Indonesia (Figure 3A). As regards the change trend from 1990 to 2019, the ASYRs in all 204 countries and territories decreased (Figure 3B).

The ASYRs due to moderate vision loss, severe vision loss, blindness, and presbyopia at the national level are listed in Supplementary Table 3. The ASYRs were the highest in Oman in terms of moderate (193.16, 95% UI: 117.24 to 303.26; per 100,000 population; Supplementary Figure 3) and severe (187.25, 95% UI: 125.26 to 272.05; per 100,000 population; Supplementary Figure 4) vision loss, followed by India. China showed the largest increment for moderate (EAPC = 0.44, 95% UI: 0.31 to 0.56; Supplementary Figure 3) and severe (EAPC = 0.69, 95%UI: 0.35 to 1.04; Supplementary Figure 4) vision loss. The highest ASYRs were in South Sudan (366.46, 95% UI: 248.14 to 528.22; per 100,000 population; Supplementary Figure 5) for blindness and in Nepal (197.62, 95% UI: 89.26 to 378.73; per 100,000 population; Supplementary Figure 6) for presbyopia. We observed a decreasing or stable trend in the ASYRs due to blindness in all countries or territories, and the highest decreasing trends was in Equatorial Guinea (EAPC = −3.46, 95% UI: −3.64 to −3.28; Supplementary Figure 5). The highest increasing trends in ASYRs due to presbyopia were found in the Russian Federation (EAPC = 0.15, 95% UI: 0.02 to 0.28; Supplementary Figure 6).

### Relationship between estimated burden of blindness and vision loss and SDI level

**Figure 4:**
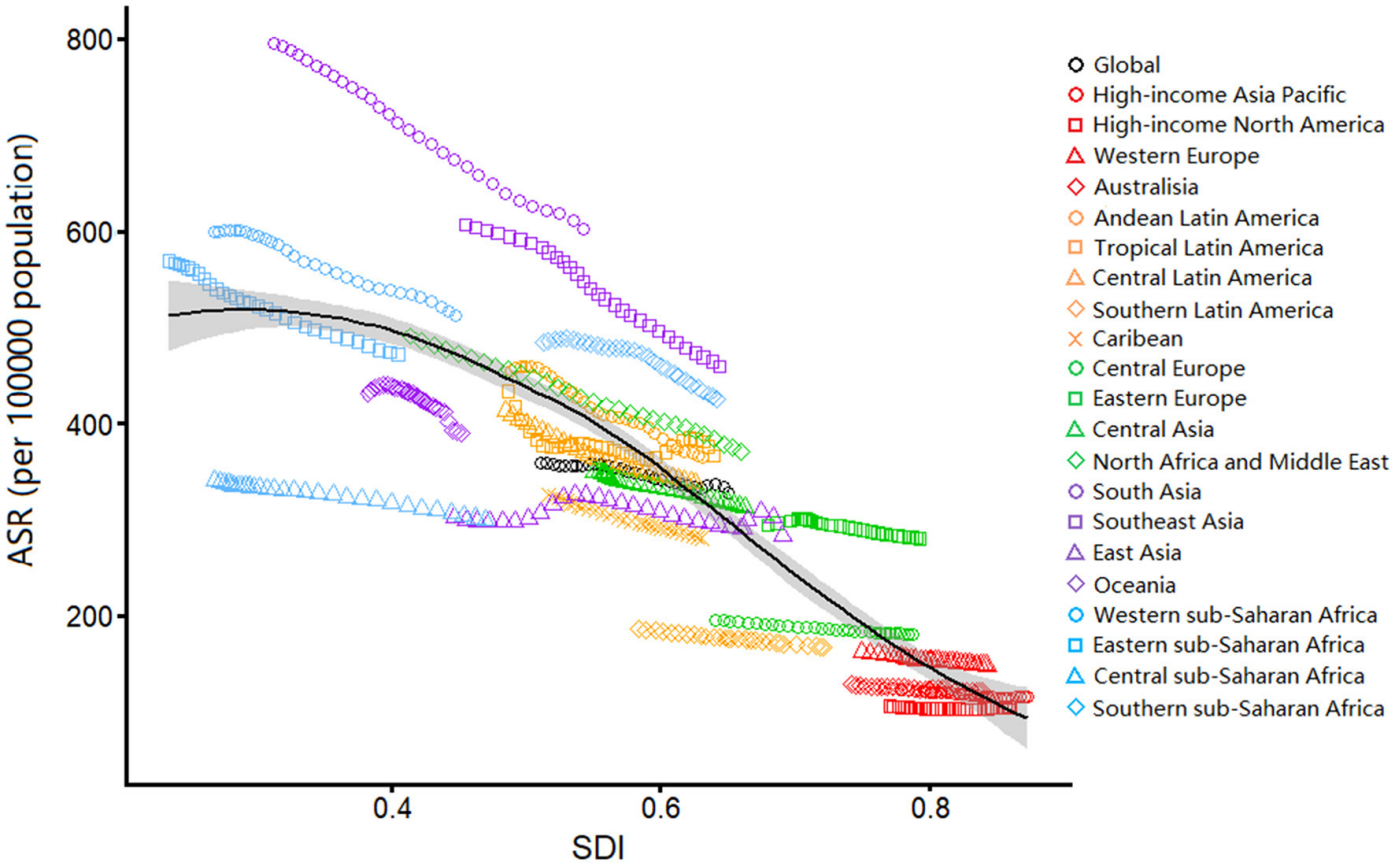
Age-standardized YLDs rates due to blindness and vision loss by SDI, 1990–2019, and expected value-based SDI. Age-standardized YLD rates are plotted for 21 geographic regions between 1990 to 2019 against their SDIs. Points in each line from left to right represents the values from 1990 to 2019. The black line represents the average expected relationship between SDI and age-standardized YLD rates due to blindness and vision loss based on values from all countries over the 1990–2019 estimation period. ASR, age-standardized rate; YLD, years lived with disability; SDI, human development index.

We described the associations between the estimated burden of blindness and vision loss and the SDI levels for each geographic region from 1990 to 2019 (Figure 4). The ASYRs and SDI levels were negatively correlated in all regions. ASYRs decreased with increased SDIs.

### Factors influencing EAPC

**Figure 5:**
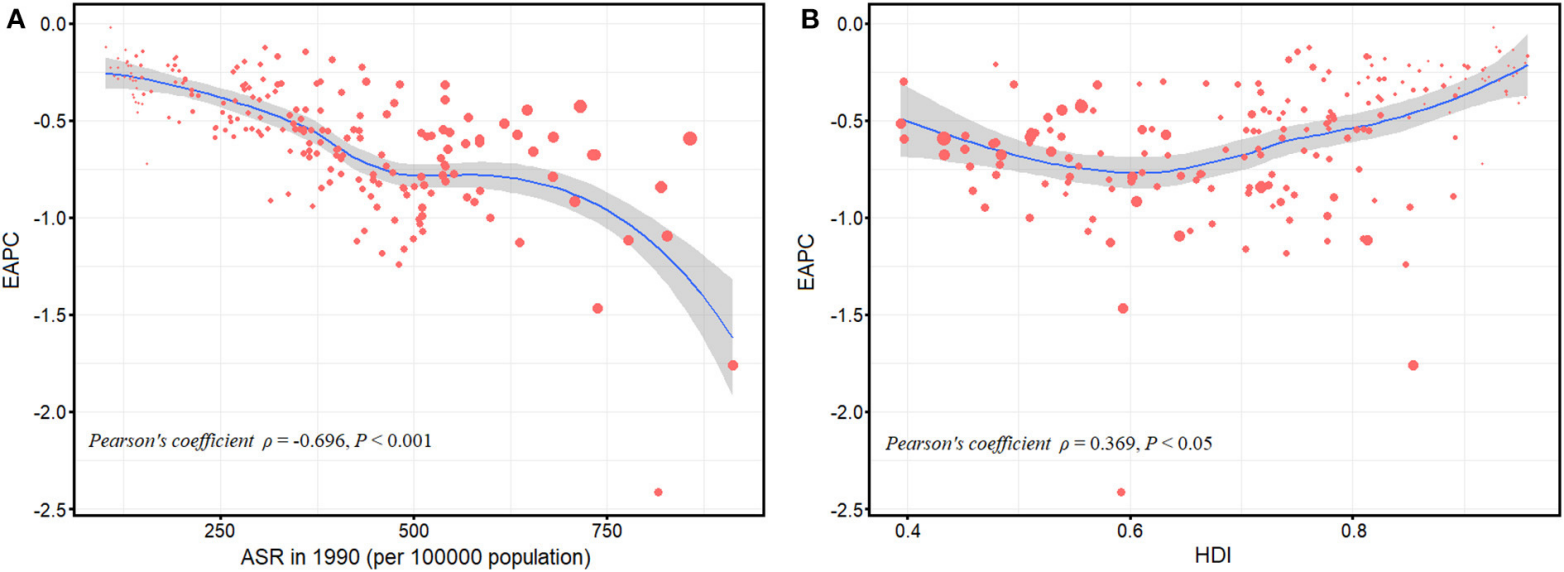
Correlation between the EAPC of age-standardized YLD rates and the age-standardized YLD rates in 1990 **(A)**; the EAPC of age-standardized YLD rates and the HDIs in 2019 **(B)**. The size of circle represents the age-standardized YLD rates in this country or territory in 2019. ASR, age-standardized rate; EAPC, estimated annual percentage change; YLD, years lived with disability; HDI, human development index.

We analyzed the relationships between EAPCs and ASYRs in 1990 and HDI in 2019 among 204 countries or territories for blindness and vision loss (Figure 5). A negative correlation was observed between the ASYRs in 1990 and the EAPCs of ASYRs (ρ = −0.696, *P* < 0.001; Figure 5A). A positive correlation was observed between the HDI in 2019 and the EAPCs of the ASYRs (ρ = 0.369, *P* < 0.05; Figure 5B).

## Discussion

This study was the first to explore the trend of the burden of blindness and vision loss and its subtypes as measured by YLD from 1990 to 2019. The ASYRs due to moderate vision loss slightly increased, whereas the ASYRs due to blindness decreased. This change may be due to the diversion of resources to treating more serious cataracts. Studies have shown that the cataract surgical coverage for eyes with visual acuity of < 3/60 is higher than that for eyes with a visual acuity of < 6/18, especially in low- and middle-income countries (9). In addition to cataract, age-related macular degeneration and diabetic retinopathy are also two important causes of blindness and vision loss. The widespread use of anti-VEGF agents to treat exudative age-related macular degeneration effectively reduces the burden of blindness and vision loss caused by it, whereas the management of diabetic retinopathy requires disproportionate resources, including the availability of ophthalmologists who are trained in laser and surgery (11).

In 2019, females had higher ASYRs due to blindness and vision loss and its subtypes than males, similar to previous findings that women have higher prevalence of blindness and vision loss than men (17–19). This difference can be attributed to two factors: physiological (e.g., women tend to live longer than men and are at greater risk for age-related eye diseases such as cataracts) (9) and social (e.g., women tend to have lower levels of education and economy, resulting in less access to health information and eye-care services; women may spend more time in the home exposed to high levels of household air pollution; time spent caring for children increases trachoma exposure; etc) factors (20). Thus, providing equal opportunities to obtain medical-system services can improve this status, especially in elderly women. Overall, ASYRs due to blindness and vision loss and its subtypes increased with age. Blindness and vision loss are the fourth leading impairments among people 70+ years old in terms of all causes of YLDs (9). Given that vision loss is associated with certain age-related eye diseases, the increase in life expectancy and aging population may increase the chance of vision damage caused by age-related eye diseases in the elderly. More health resources should be oriented toward the growing elderly populations.

Globally, ASYRs due to blindness and vision loss were most common in low-income regions, such as South Asia, Southern Sub-Saharan Africa, and Western Sub-Saharan Africa. In some South Asian countries such as India and Pakistan, where eye-health plans are insufficiently implemented, eye-health resources are limited by the heavy health needs, large backlog of people with vision loss due to huge population base, economic benefits and costs, as well as insufficient human resources for eye care (21). Different from the high-income regions, cataracts are still the most frequent cause of blindness in some underdeveloped regions (such as Sub-Saharan Africa) (22, 23). The average cataract-surgery rate in Sub-Saharan Africa countries is 442 operations/million person/year, far less than the 2,000/million persons/year proposed by VISION 2020 (22, 24). Greater government investment in medical infrastructure, training of eye care practitioners, and strategies for cataract surgery are needed.

According to the estimates from the GBD 2019, the most significant increase in moderate and severe vision loss occurred in China, and the most significant increase in presbyopia occurred in the Russian Federation. In the early 1990s, the Soviet Union disintegrated, Russian Federation's economy was in recession, and a large middle-aged population migrated to Western European countries (25). The loss of the middle-aged population means a relative increase in the elderly population, which may increase the burden of presbyopia. The increasing phenomenon in China is probably related to the increase in the prevalence of diabetic retinopathy since 1990 (26). The rapid increase in metabolic diseases related to lifestyle changes has increased the burden of eye diseases, especially diabetes, which has a great risk of developing into diabetic retinopathy (27). Moreover, uncorrected refractive errors were the first and second causes of moderate and severe vision loss, and blindness, respectively. Notably, myopia has become the greatest burden of refractive errors (28). In China, the rates of myopia and high myopia among children and adolescents have increased significantly over the past few decades (29), so the vision loss caused by myopia-related complications and uncorrected myopia cannot be ignored. Studies have shown that myopia macular degeneration is also an important cause of vision loss in East Asia (30). Disseminating scientific knowledge about eye health, raising public awareness on vision health, and promoting early screening and correction of adolescent refractive errors through professional ophthalmological institutions are important.

ASYRs due to blindness and vision loss showed an overall decreasing trend with increased SDIs. In 2019, ASYRs were highest in the low-SDI regions and lowest in the high-SDI regions. Generally, a higher socioeconomic status is related to higher health expenditures and higher level of eye health (21, 31, 32). Low-income countries may allocate a small portion of their government spending to health-care resources. Research estimates indicate that by 2040, health expenditures in many low-income countries will remain low, and only 3% low-income countries will meet the Chatham House goal of 5% of GDP (30). Due to the reduced allocation of health-expenditure resources, eye-care services are unaffordable and inaccessible in low-income countries, where more people with uncorrected refractive errors lack access to cataract surgery (21). The correction rate of glasses for presbyopia in developing countries ranges from 6% to 45% (33). Moreover, low education level may affect the level of eye-care services sought. Data from the UK shows that people with no educational qualifications are nearly twice as likely to suffer from moderate to severe vision impairment and blindness as those with university-level or other professional qualifications (34). Among the Korean elderly population, the lifetime use of eye care is affected by the level of education (35). Educational intervention can increase the acceptance and willingness to pay for cataract surgery by targeting the rural population (36). Therefore, more wealth must be invested in social welfare and educational interventions and consulting services must be provided for people with low education levels.

We also found that variations in ASYRs due to blindness and vision loss from 1990 to 2019 were significantly positively correlated with HDI in 2019. However, given that the EAPC is almost below 0, countries with low HDI in 2019 are more likely to see their burden reduced by more. These results can be explained as follows: (1) as a result of the release of the Global Action Plan, underdeveloped countries have increased their investment in eye care and made some progress; and (2) many countries with low HDI have seen significant increases in development assistance for health, which has helped provide essential services for priority diseases.

Some limitations inevitably exist in this study. First, GBD data were calculated based on existing data of various countries by using algorithms. The accuracy of data depends to a large extent on the quality and quantity of the data used in the algorithm, which could affect the accuracy of the estimated burden (37). Second, data are sparse in representative regions and ethnic groups in many countries and age groups. Sparse data limit the certainty of estimation of temporal trends and age patterns, especially among children and young people (38). Despite these limitations, the GBD remains the most standardized and accurate system available for assessing the burden of disease across time, location, and different diseases and injuries.

## Conclusion

The burden of blindness and vision loss varies by region, country, gender, age, and socioeconomic development. Globally, although ASYRs due to blindness and vision loss slightly decreased from 1990 to 2019, they will continue to cause great losses in healthy life in the future, especially in the elderly. To combat the high burden of blindness and vision loss, especially in low-SDI and low-middle SDI regions, increasing investment in eye-care services and allocating eye care resources rationally can be considered. We also need novel and cheaper treatments to tackle conditions that affect large numbers of individuals, such as age-related macular degeneration and diabetic retinopathy.

## Data availability statement

The original contributions presented in the study are included in the article/Supplementary material, further inquiries can be directed to the corresponding author.

## Author contributions

SL, SY, and JWu: study design. SL, YZ, SY, and JL: data collection. JWa, XHu, XHo, and JWu: data analysis. SL, EY, and JH: drafting the manuscript. SL, EY, DN, and XHo: revising the manuscript. All authors listed have made a substantial, direct, and intellectual contribution to the work and approved it for publication.

## Conflict of interest

The authors declare that the research was conducted in the absence of any commercial or financial relationships that could be construed as a potential conflict of interest.

## Publisher's note

All claims expressed in this article are solely those of the authors and do not necessarily represent those of their affiliated organizations, or those of the publisher, the editors and the reviewers. Any product that may be evaluated in this article, or claim that may be made by its manufacturer, is not guaranteed or endorsed by the publisher.

## Supplementary material

Supplementary Figure 1 The EAPC of age-standardized YLD rates due to moderate vision loss **(A)**, severe vision loss **(B)**, blindness **(C)**, and presbyopia **(D)** at the global and regional levels. EAPC, estimated annual percentage change; YLD, years lived with disability; SDI, human development index.

Supplementary Figure 2 The YLD rate due to moderate vision loss **(A)**, severe vision loss **(B)**, blindness **(C)**, and presbyopia **(D)** of different age groups globally. YLD, years lived with disability.

Supplementary Figure 3 The global burden due to moderate vision loss in 204 countries or territories. **(A)** The age-standardized YLD rate due to moderate vision loss in 2019. **(B)** The EAPC of age-standardized YLD rate due to moderate vision loss from 1990 to 2019. ASR, age-standardized rate; EAPC, estimated annual percentage change; YLD, years lived with disability.

Supplementary Figure 4 The global burden due to severe vision loss in 204 countries or territories. **(A)** The age-standardized YLD rate due to severe vision loss in 2019. **(B)** The EAPC of age-standardized YLD rate due to severe vision loss from 1990 to 2019. ASR, age-standardized rate; EAPC, estimated annual percentage change; YLD, years lived with disability.

Supplementary Figure 5 The global burden due to blindness in 204 countries or territories. **(A)** The age-standardized YLD rate due to blindness in 2019. **(B)** The EAPC of age-standardized YLD rate due to blindness from 1990 to 2019. ASR, age-standardized rate; EAPC, estimated annual percentage change; YLD, years lived with disability.

Supplementary Figure 6 The global burden due to presbyopia in 204 countries or territories. **(A)** The age-standardized YLD rate due to presbyopia in 2019. **(B)** The EAPC of age-standardized YLD rate due to presbyopia from 1990 to 2019. ASR, age-standardized rate; EAPC, estimated annual percentage change; YLD, years lived with disability.

## Abbreviations

YLDs: years lived with disability
ASR: age-standardized rate
ASYRs: age-standardized YLD rates
SDI: sociodemographic index
HDIs: human development indices
GBD: Global Burden of Disease
EAPC: estimated annual percentage change
DWs: disability weights
UI: uncertainty interval
CI: confidence interval.
