# Supplementary figures and images for: Global, regional, and national years lived with disability due to blindness and vision loss from 1990 to 2019: Findings from the Global Burden of Disease Study 2019
Source: Front Public Health. 2022 Oct 28;10:1033495. doi: 10.3389/fpubh.2022.1033495 (PMC9650182; doi:10.3389/fpubh.2022.1033495)

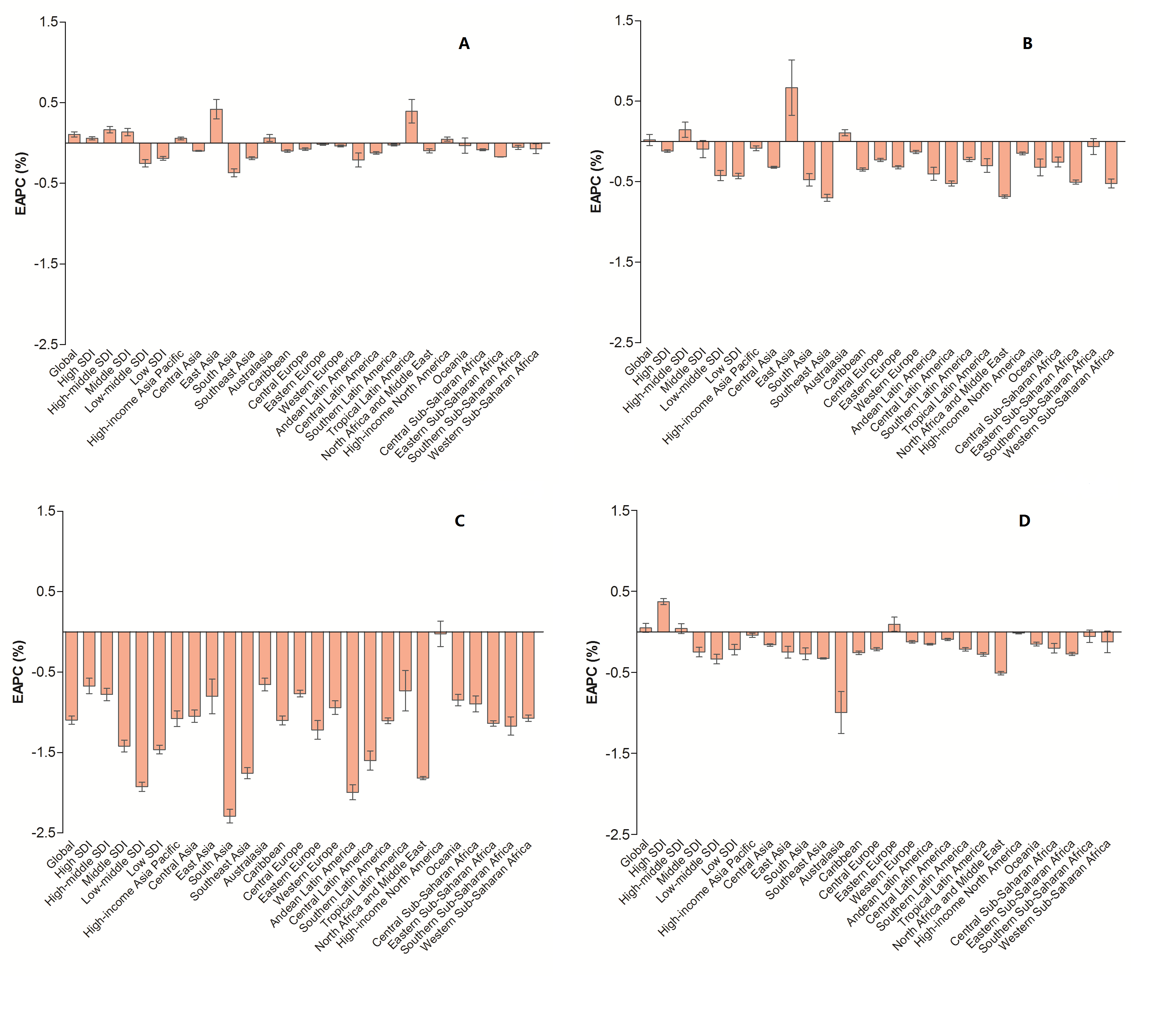

Supplement: Supplementary Figure 1 — The EAPC of age-standardized YLD rates due to moderate vision loss (A), severe vision loss (B), blindness (C), and presbyopia (D) at the global and regional levels. EAPC, estimated annual percentage change; YLD, years lived with disability; SDI, human development index. [file Image_1.TIF]

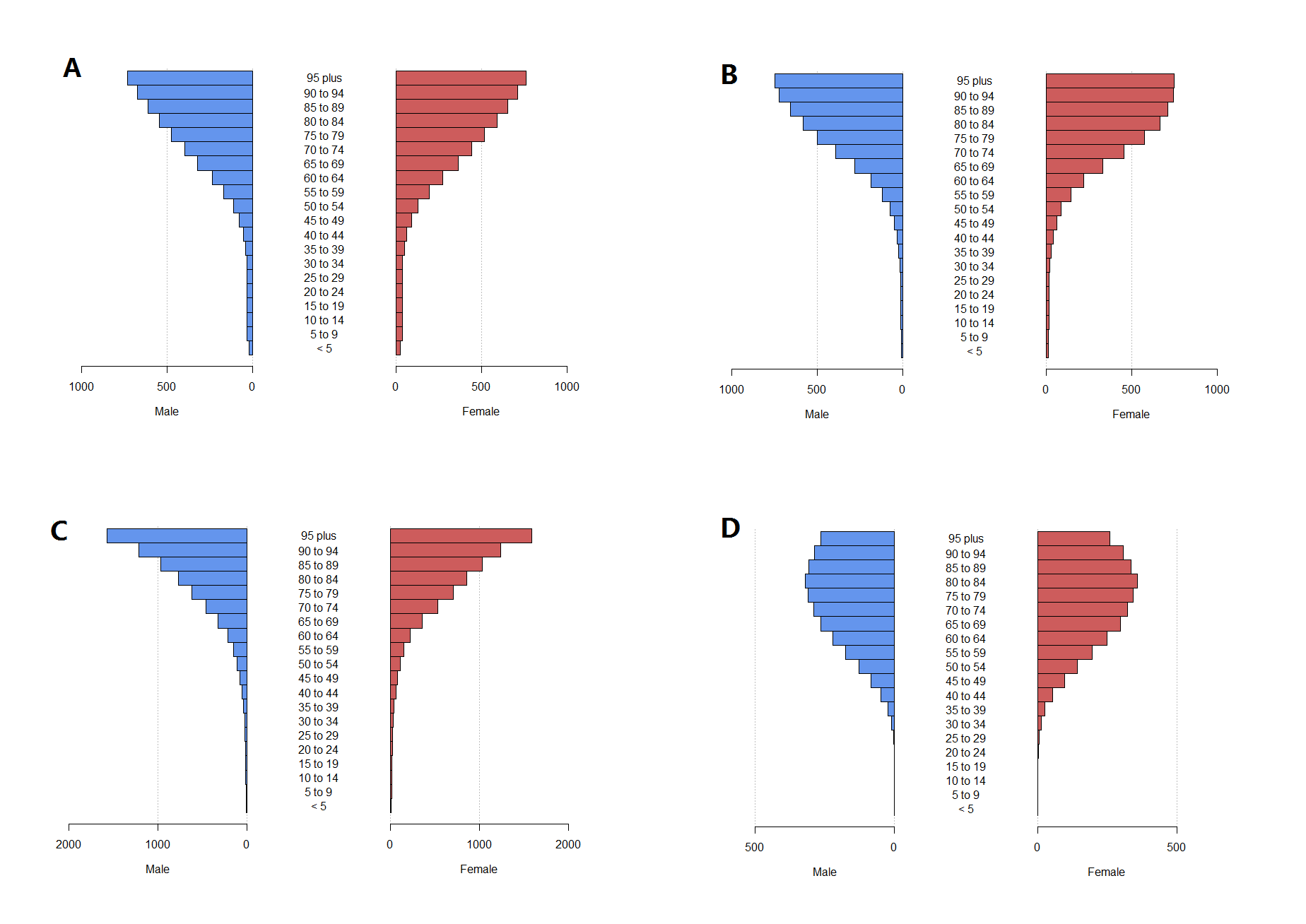

Supplement: Supplementary Figure 2 — The YLD rate due to moderate vision loss (A), severe vision loss (B), blindness (C), and presbyopia (D) of different age groups globally. YLD, years lived with disability. [file Image_2.TIF]

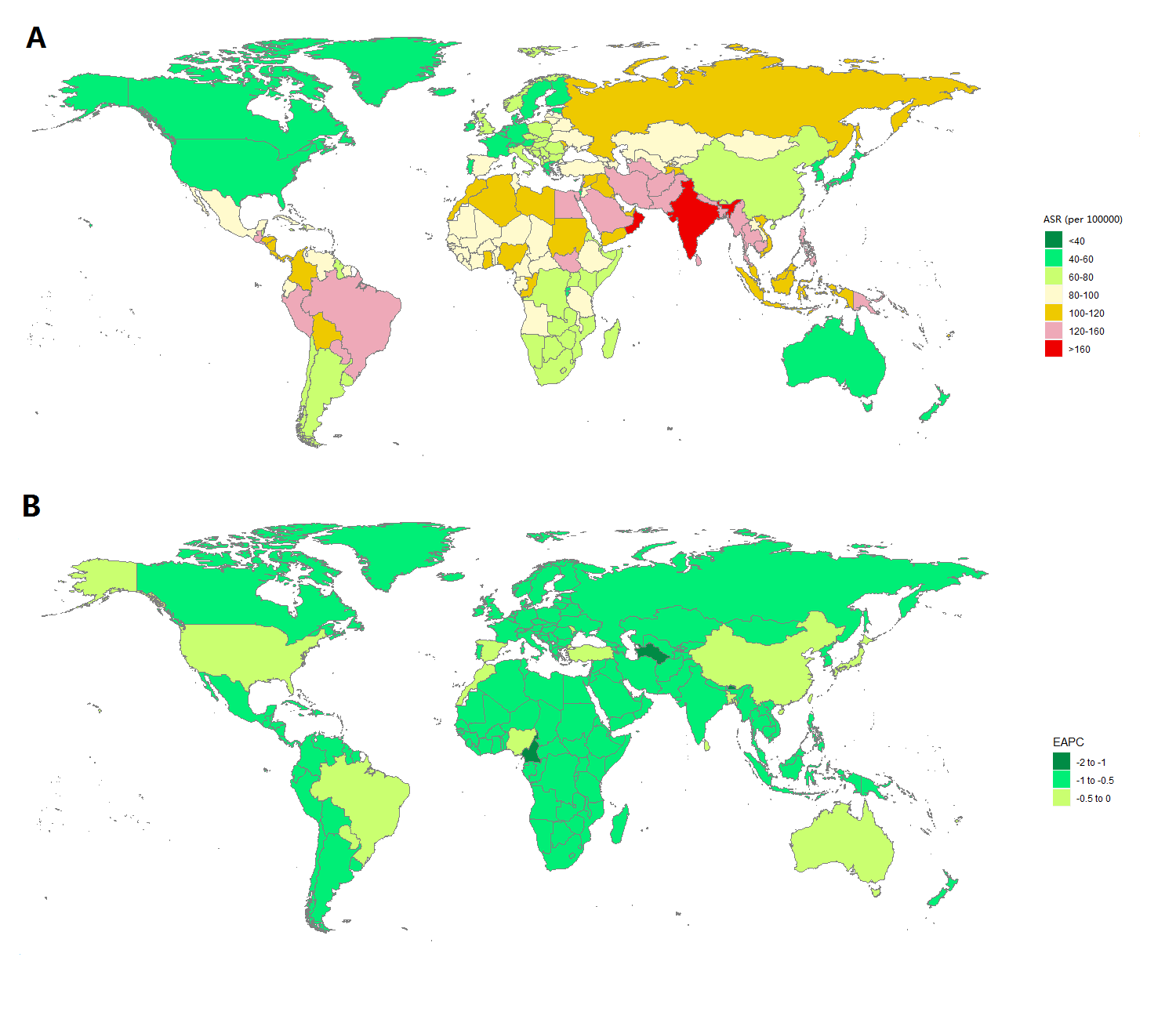

Supplement: Supplementary Figure 3 — The global burden due to moderate vision loss in 204 countries or territories. (A) The age-standardized YLD rate due to moderate vision loss in 2019. (B) The EAPC of age-standardized YLD rate due to moderate vision loss from 1990 to 2019. ASR, age-standardized rate; EAPC, estimated annual percentage change; YLD, years lived with disability. [file Image_3.TIF]

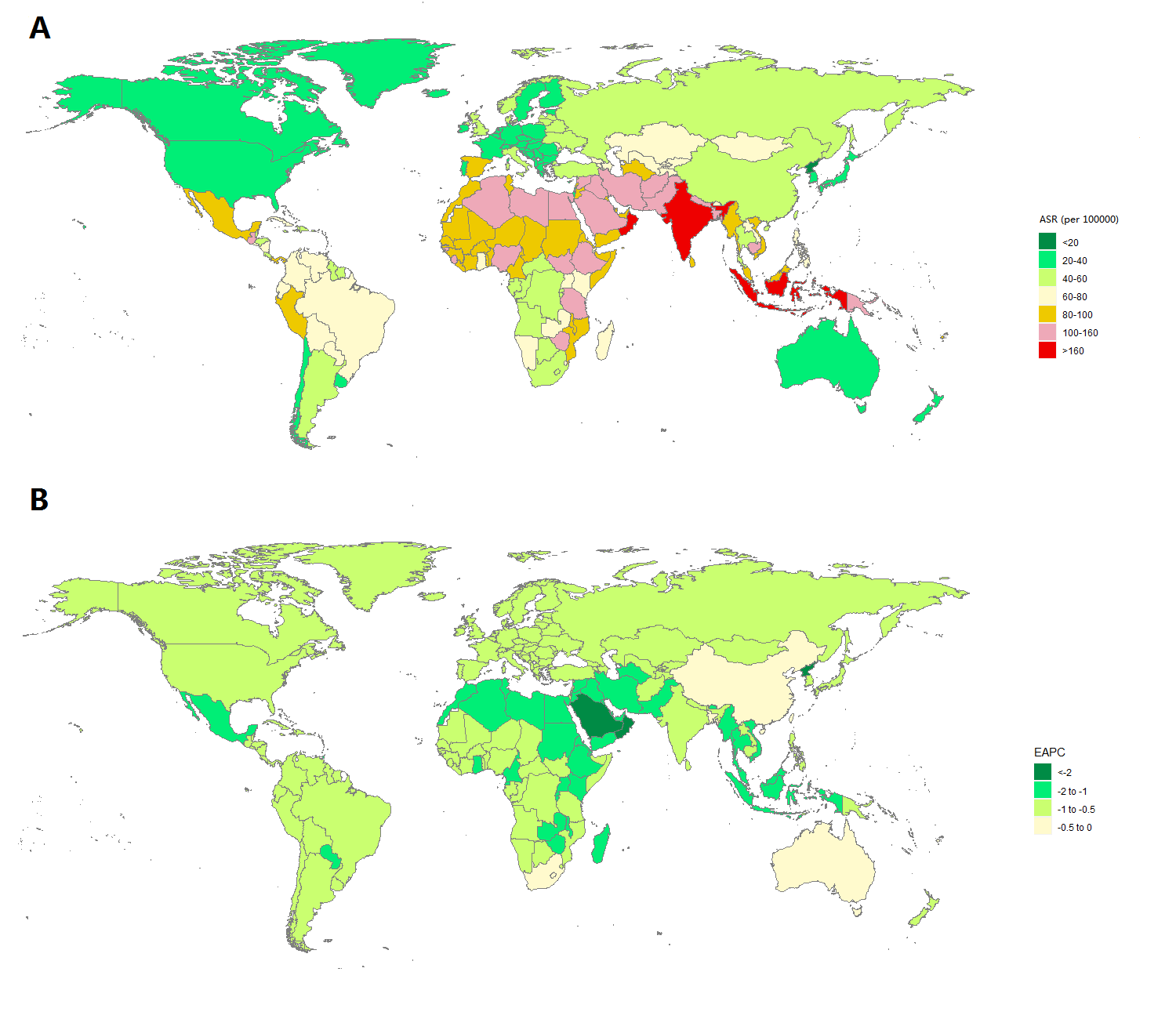

Supplement: Supplementary Figure 4 — The global burden due to severe vision loss in 204 countries or territories. (A) The age-standardized YLD rate due to severe vision loss in 2019. (B) The EAPC of age-standardized YLD rate due to severe vision loss from 1990 to 2019. ASR, age-standardized rate; EAPC, estimated annual percentage change; YLD, years lived with disability. [file Image_4.TIF]

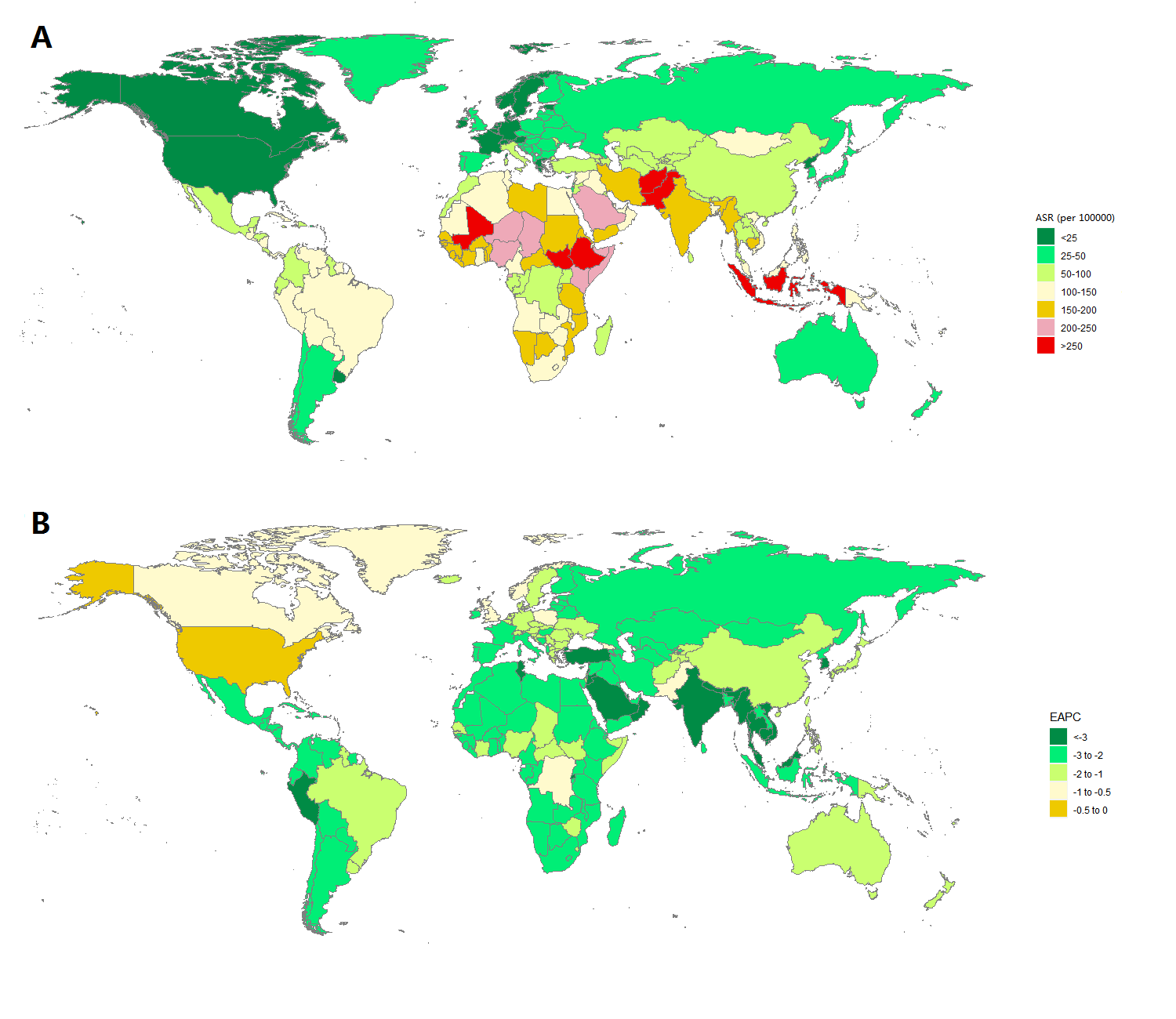

Supplement: Supplementary Figure 5 — The global burden due to blindness in 204 countries or territories. (A) The age-standardized YLD rate due to blindness in 2019. (B) The EAPC of age-standardized YLD rate due to blindness from 1990 to 2019. ASR, age-standardized rate; EAPC, estimated annual percentage change; YLD, years lived with disability. [file Image_5.TIF]

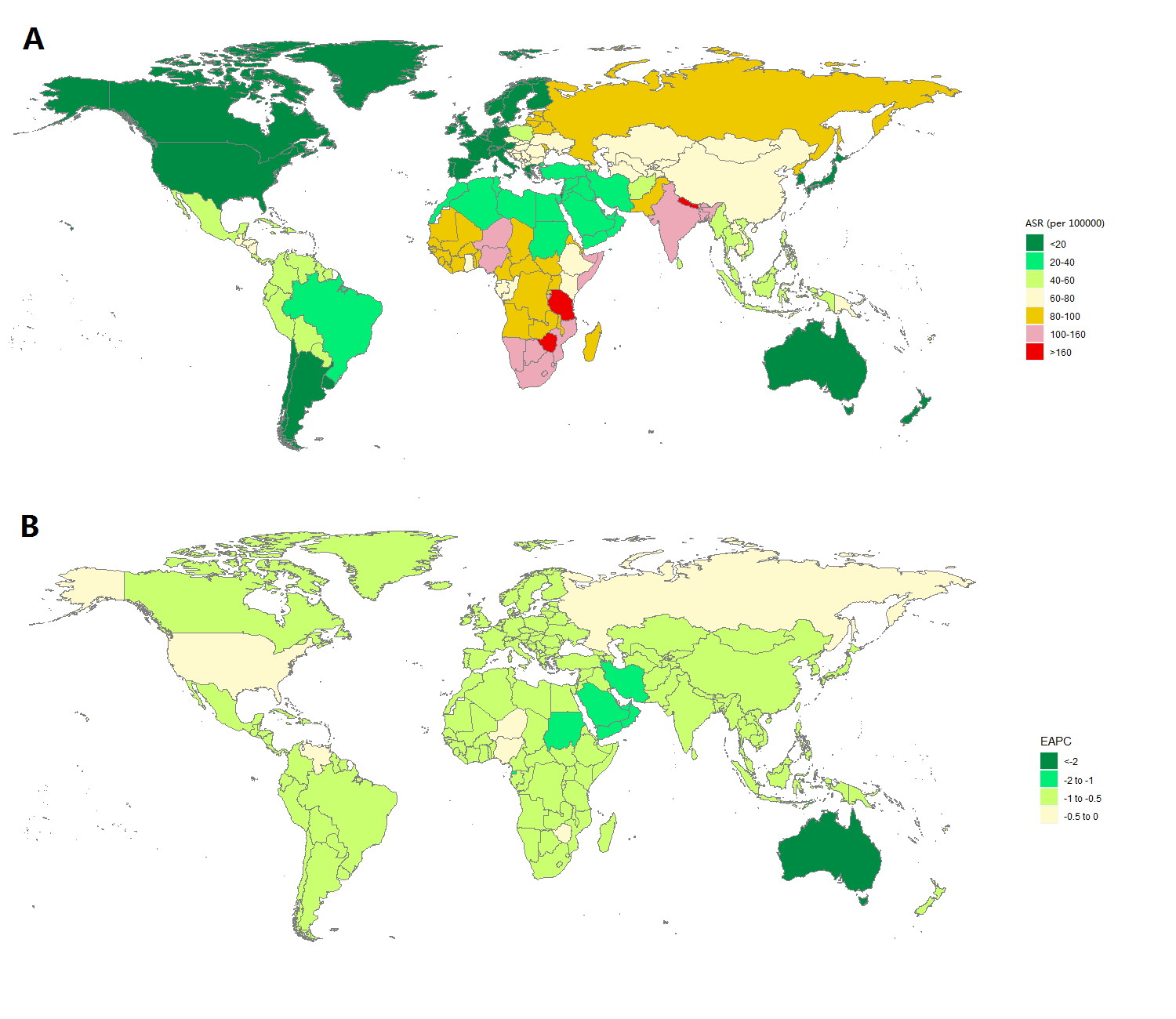

Supplement: Supplementary Figure 6 — The global burden due to presbyopia in 204 countries or territories. (A) The age-standardized YLD rate due to presbyopia in 2019. (B) The EAPC of age-standardized YLD rate due to presbyopia from 1990 to 2019. ASR, age-standardized rate; EAPC, estimated annual percentage change; YLD, years lived with disability. [file Image_6.TIF]
