# Supplementary material for: Global, regional, and national years lived with disability due to blindness and vision loss from 1990 to 2019: Findings from the Global Burden of Disease Study 2019
Source: Front Public Health. 2022 Oct 28;10:1033495. doi: 10.3389/fpubh.2022.1033495 (PMC9650182; doi:10.3389/fpubh.2022.1033495)
Supplement: Supplementary file 7 [file Table_1.docx]

**Supplementary Table 1. Age-standardized rates of years lived with disability due to moderate vision loss, severe vision loss, blindness and presbyopia in 2019 and their temporal trend from 1990 to 2019 at the global and regional levels**

|  | Moderate vision loss（per 100000 population） | | |  | Severe vision loss（per 100000 population） | | |  | Blindness（per 100000 population） | | |  | Presbyopia（per 100000 population） | | |
| --- | --- | --- | --- | --- | --- | --- | --- | --- | --- | --- | --- | --- | --- | --- | --- |
|  | ASYRs in 1990 | ASYRs in 2019 | EAPC (1990-2019, 95% UI) |  | ASYRs in 1990 | ASYRs in 2019 | EAPC  (1990-2019, 95% UI) |  | ASYRs in 1990 | ASYRs in 2019 | EAPC  (1990-2019, 95% UI) |  | ASYRs in 1990 | ASYRs in 2019 | EAPC  (1990-2019, 95% UI) |
| **Global** | 94.26 | 95.81 | 0.11 (0.08 ‒ 0.14) |  | 76.75 | 74.86 | 0.02 (-0.05 ‒ 0.09) |  | 129.69 | 95.03 | -1.10 (-1.15 ‒ -1.05) |  | 58.76 | 62.27 | 0.05 (-0.01 ‒ 0.11) |
| **Sex** |  |  |  |  |  |  |  |  |  |  |  |  |  |  |  |
| Male | 90.43 | 90.61 | 0.06 (0.03 ‒ 0.09) |  | 73.58 | 69.40 | -0.11 (-0.18 ‒ -0.04) |  | 131.29 | 91.83 | -1.27 (-1.32 ‒ -1.22) |  | 57.09 | 58.88 | -0.05 (-0.11 ‒ 0.01) |
| Female | 97.83 | 100.65 | 0.15 (0.12 ‒ 0.19) |  | 79.80 | 79.81 | 0.11 (0.04 ‒ 0.19) |  | 128.73 | 97.68 | -0.97 (-1.02 ‒ -0.92) |  | 60.34 | 65.37 | 0.13 (0.07 ‒ 0.18) |
| **Socio-demographic index** |  |  |  |  |  |  |  |  |  |  |  |  |  |  |  |
| High SDI | 53.05 | 53.86 | 0.06 (0.04 ‒ 0.08) |  | 36.37 | 35.01 | -0.12 (-0.14 ‒ -0.10) |  | 32.50 | 26.95 | -0.67 (-0.77 ‒ -0.58) |  | 11.55 | 12.98 | 0.37 (0.34 ‒ 0.41) |
| High-middle SDI | 84.78 | 87.57 | 0.17 (0.13 ‒ 0.21) |  | 56.93 | 56.14 | 0.15 (0.05 ‒ 0.24) |  | 87.10 | 69.21 | -0.78 (-0.86 ‒ -0.70) |  | 57.08 | 60.61 | 0.04 (-0.02 ‒ 0.10) |
| Middle SDI | 105.39 | 108.45 | 0.14 (0.09 ‒ 0.18) |  | 90.79 | 84.89 | -0.10 (-0.20 ‒ 0.01) |  | 187.55 | 123.20 | -1.42 (-1.49 ‒ -1.35) |  | 72.31 | 70.21 | -0.25 (-0.31 ‒ -0.19) |
| Low-middle SDI | 153.88 | 137.99 | -0.25 (-0.30 ‒ -0.21) |  | 143.48 | 123.84 | -0.43 (-0.49 ‒ -0.36) |  | 254.74 | 148.86 | -1.93 (-1.98 ‒ -1.87) |  | 99.35 | 93.48 | -0.34 (-0.39 ‒ -0.28) |
| Low SDI | 129.24 | 120.41 | -0.19 (-0.21 ‒ -0.17) |  | 140.29 | 122.22 | -0.43 (-0.46 ‒ -0.40) |  | 278.79 | 187.30 | -1.46 (-1.52 ‒ -1.41) |  | 110.61 | 106.67 | -0.22 (-0.28 ‒ -0.16) |
| **Geographic region** |  |  |  |  |  |  |  |  |  |  |  |  |  |  |  |
| High-income Asia Pacific | 47.75 | 48.25 | 0.06 (0.04 ‒ 0.08) |  | 36.12 | 35.69 | -0.09 (-0.12 ‒ -0.06) |  | 36.47 | 26.74 | -1.08 (-1.18 ‒ -0.98) |  | 4.80 | 4.81 | -0.04 (-0.07 ‒ -0.02) |
| Central Asia | 102.64 | 99.65 | -0.10 (-0.11 ‒ -0.09) |  | 78.14 | 70.88 | -0.32 (-0.33 ‒ -0.31) |  | 97.34 | 74.00 | -1.05 (-1.12 ‒ -0.97) |  | 71.29 | 68.61 | -0.16 (-0.18 ‒ -0.15) |
| East Asia | 68.71 | 77.05 | 0.42 (0.30 ‒ 0.54) |  | 42.18 | 43.79 | 0.67 (0.33 ‒ 1.01) |  | 112.60 | 85.55 | -0.80 (-1.02 ‒ -0.59) |  | 79.06 | 77.54 | -0.25 (-0.32 ‒ -0.18) |
| South Asia | 191.50 | 166.77 | -0.37 (-0.42 ‒ -0.32) |  | 187.61 | 160.29 | -0.48 (-0.55 ‒ -0.40) |  | 297.83 | 160.01 | -2.29 (-2.38 ‒ -2.21) |  | 119.29 | 114.89 | -0.27 (-0.34 ‒ -0.20) |
| Southeast Asia | 129.23 | 123.04 | -0.19 (-0.20 ‒ -0.17) |  | 128.75 | 105.93 | -0.70 (-0.74 ‒ -0.66) |  | 294.59 | 180.92 | -1.76 (-1.83 ‒ -1.69) |  | 54.73 | 49.63 | -0.33 (-0.34 ‒ -0.32) |
| Australasia | 56.45 | 57.10 | 0.06 (0.02 ‒ 0.11) |  | 29.33 | 29.81 | 0.11 (0.07 ‒ 0.15) |  | 33.58 | 27.22 | -0.66 (-0.74 ‒ -0.58) |  | 9.25 | 6.97 | -1.00 (-1.25 ‒ -0.74) |
| Caribbean | 86.90 | 84.38 | -0.10 (-0.11 ‒ -0.09) |  | 58.18 | 52.81 | -0.35 (-0.37 ‒ -0.33) |  | 124.49 | 90.50 | -1.10 (-1.16 ‒ -1.05) |  | 56.82 | 53.11 | -0.26 (-0.28 ‒ -0.24) |
| Central Europe | 62.69 | 61.39 | -0.08 (-0.09 ‒ -0.06) |  | 30.41 | 28.67 | -0.23 (-0.25 ‒ -0.21) |  | 38.47 | 31.04 | -0.77 (-0.81 ‒ -0.73) |  | 63.96 | 60.02 | -0.21 (-0.23 ‒ -0.19) |
| Eastern Europe | 102.42 | 101.42 | -0.02 (-0.03 ‒ 0.00) |  | 60.59 | 55.49 | -0.32 (-0.34 ‒ -0.30) |  | 59.14 | 44.56 | -1.22 (-1.33 ‒ -1.10) |  | 72.31 | 78.98 | 0.09 (0.01 ‒ 0.18) |
| Western Europe | 66.96 | 65.86 | -0.04 (-0.05 ‒ -0.02) |  | 46.91 | 44.54 | -0.13 (-0.15 ‒ -0.11) |  | 42.93 | 32.72 | -0.94 (-1.03 ‒ -0.86) |  | 5.53 | 5.35 | -0.12 (-0.14 ‒ -0.11) |
| Andean Latin America | 128.70 | 124.65 | -0.21 (-0.30 ‒ -0.12) |  | 91.05 | 83.44 | -0.40 (-0.48 ‒ -0.32) |  | 184.07 | 108.17 | -2.00 (-2.09 ‒ -1.90) |  | 51.74 | 49.50 | -0.15 (-0.16 ‒ -0.14) |
| Central Latin America | 107.63 | 102.82 | -0.12 (-0.14 ‒ -0.11) |  | 97.10 | 84.60 | -0.52 (-0.55 ‒ -0.49) |  | 150.15 | 92.58 | -1.6 (-1.72 ‒ -1.48) |  | 58.45 | 57.46 | -0.09 (-0.11 ‒ -0.08) |
| Southern Latin America | 79.11 | 77.95 | -0.03 (-0.04 ‒ -0.01) |  | 51.44 | 47.60 | -0.22 (-0.25 ‒ -0.20) |  | 49.40 | 35.44 | -1.10 (-1.14 ‒ -1.07) |  | 6.45 | 6.08 | -0.22 (-0.24 ‒ -0.19) |
| Tropical Latin America | 124.73 | 120.55 | 0.40 (0.25 ‒ 0.54) |  | 83.57 | 73.52 | -0.30 (-0.38 ‒ -0.21) |  | 184.87 | 134.88 | -0.73 (-0.98 ‒ -0.48) |  | 40.61 | 37.54 | -0.28 (-0.30 ‒ -0.26) |
| North Africa and Middle East | 119.26 | 114.90 | -0.1 (-0.12 ‒ -0.07) |  | 117.49 | 96.17 | -0.68 (-0.70 ‒ -0.66) |  | 215.50 | 126.92 | -1.82 (-1.84 ‒ -1.80) |  | 39.00 | 33.61 | -0.51 (-0.53 ‒ -0.49) |
| High-income North America | 43.33 | 43.67 | 0.05 (0.02 ‒ 0.07) |  | 32.16 | 30.71 | -0.15 (-0.17 ‒ -0.13) |  | 22.63 | 22.71 | -0.02 (-0.18 ‒ 0.14) |  | 8.37 | 8.35 | -0.01 (-0.03 ‒ 0.00) |
| Oceania | 133.13 | 133.28 | -0.03 (-0.13 ‒ 0.06) |  | 102.28 | 94.10 | -0.32 (-0.43 ‒ -0.22) |  | 131.40 | 100.45 | -0.85 (-0.92 ‒ -0.78) |  | 64.54 | 61.82 | -0.15 (-0.17 ‒ -0.13) |
| Central Sub-Saharan Africa | 82.21 | 80.37 | -0.08 (-0.10 ‒ -0.07) |  | 47.92 | 43.95 | -0.26 (-0.32 ‒ -0.20) |  | 118.32 | 88.34 | -0.90 (-1.00 ‒ -0.8) |  | 92.14 | 86.87 | -0.20 (-0.26 ‒ -0.14) |
| Eastern Sub-Saharan Africa | 85.67 | 81.46 | -0.17 (-0.17 ‒ -0.17) |  | 114.78 | 98.5 | -0.51 (-0.53 ‒ -0.48) |  | 260.87 | 192.32 | -1.14 (-1.17 ‒ -1.10) |  | 108.16 | 99.76 | -0.27 (-0.29 ‒ -0.25) |
| Southern Sub-Saharan Africa | 64.53 | 63.13 | -0.05 (-0.08 ‒ -0.03) |  | 59.60 | 56.54 | -0.06 (-0.16 ‒ 0.04) |  | 208.36 | 149.24 | -1.17 (-1.28 ‒ -1.06) |  | 152.22 | 157.32 | -0.06 (-0.13 ‒ 0.02) |
| Western Sub-Saharan Africa | 105.52 | 104.19 | -0.07 (-0.13 ‒ -0.01) |  | 126.60 | 110.45 | -0.52 (-0.58 ‒ -0.47) |  | 274.58 | 200.34 | -1.07 (-1.11 ‒ -1.03) |  | 92.43 | 97.07 | -0.12 (-0.26 ‒ 0.01) |
| YLDs, years lived with disability; ASYR, age-standardized YLD rate; EAPC, estimated annual percentage change; UI, uncertainty interval. | | | | | | | | | | | | | | | |
