# Supplementary material for: Global, regional, and national years lived with disability due to blindness and vision loss from 1990 to 2019: Findings from the Global Burden of Disease Study 2019
Source: Front Public Health. 2022 Oct 28;10:1033495. doi: 10.3389/fpubh.2022.1033495 (PMC9650182; doi:10.3389/fpubh.2022.1033495)
Supplement: Supplementary file 8 [file Table_2.docx]

**Supplementary Table 2. Age-standardized rates of years lived with disability due to blindness and vision loss in 2019 and their temporal trend from 1990 to 2019 at the national level**

|  | ASYRs in 1990  (per 100000 population, 95% UI) | ASYRs in 2019  (per 100000 population, 95% UI) | EAPC  (1990-2019, 95% UI) |
| --- | --- | --- | --- |
| Afghanistan | 680.68 (473.34 ‒ 933.97) | 577.73 (398.68 ‒ 795.02) | -0.59 (-0.74 ‒ -0.43) |
| Albania | 212.69 (138.95 ‒ 316.75) | 193.06 (123.34 ‒ 290.75) | -0.37 (-0.39 ‒ -0.35) |
| Algeria | 491.13 (342.67 ‒ 675.57) | 377.48 (259.90 ‒ 521.33) | -0.89 (-0.92 ‒ -0.86) |
| American Samoa | 311.08 (210.43 ‒ 442.90) | 277.35 (187.64 ‒ 402.62) | -0.38 (-0.39 ‒ -0.36) |
| Andorra | 129.45 (88.33 ‒ 182.09) | 122.16 (82.57 ‒ 172.00) | -0.19 (-0.21 ‒ -0.17) |
| Angola | 428.09 (290.43 ‒ 602.70) | 340.85 (229.75 ‒ 494.01) | -0.8 (-0.85 ‒ -0.76) |
| Antigua and Barbuda | 261.83 (177.11 ‒ 375.52) | 227.31 (152.54 ‒ 329.46) | -0.48 (-0.5 ‒ -0.46) |
| Argentina | 192.00 (131.87 ‒ 265.84) | 173.73 (119.14 ‒ 242.81) | -0.3 (-0.33 ‒ -0.28) |
| Armenia | 344.69 (233.15 ‒ 491.09) | 301.58 (202.29 ‒ 438.55) | -0.52 (-0.54 ‒ -0.5) |
| Australia | 126.99 (85.96 ‒ 179.48) | 120.40 (81.18 ‒ 169.67) | -0.14 (-0.17 ‒ -0.10) |
| Austria | 135.99 (93.19 ‒ 191.03) | 125.11 (84.88 ‒ 175.26) | -0.27 (-0.29 ‒ -0.26) |
| Azerbaijan | 347.89 (237.07 ‒ 492.38) | 311.16 (209.27 ‒ 447.72) | -0.45 (-0.50 ‒ -0.39) |
| Bahamas | 253.81 (172.29 ‒ 359.37) | 226.62 (152.60 ‒ 324.08) | -0.41 (-0.45 ‒ -0.38) |
| Bahrain | 452.33 (313.70 ‒ 618.61) | 348.33 (240.57 ‒ 483.11) | -0.95 (-0.99 ‒ -0.91) |
| Bangladesh | 633.85 (433.29 ‒ 889.50) | 506.09 (341.45 ‒ 720.01) | -0.58 (-0.67 ‒ -0.48) |
| Barbados | 148.34 (97.00 ‒ 221.41) | 135.62 (87.66 ‒ 207.84) | -0.28 (-0.30 ‒ -0.26) |
| Belarus | 292.65 (192.06 ‒ 441.39) | 263.11 (170.24 ‒ 398.21) | -0.38 (-0.39 ‒ -0.37) |
| Belgium | 136.50 (93.65 ‒ 190.29) | 124.93 (85.44 ‒ 175.27) | -0.30 (-0.31 ‒ -0.28) |
| Belize | 299.66 (206.23 ‒ 423.92) | 252.04 (169.96 ‒ 362.70) | -0.56 (-0.59 ‒ -0.53) |
| Benin | 534.58 (369.64 ‒ 748.01) | 435.90 (298.12 ‒ 614.28) | -0.7 (-0.72 ‒ -0.67) |
| Bermuda | 242.56 (162.67 ‒ 347.64) | 208.37 (139.95 ‒ 305.74) | -0.54 (-0.57 ‒ -0.51) |
| Bhutan | 435.65 (294.66 ‒ 634.57) | 325.45 (213.98 ‒ 488.23) | -1.07 (-1.12 ‒ -1.01) |
| Bolivia (Plurinational State of) | 459.27 (321.40 ‒ 632.62) | 372.33 (257.06 ‒ 519.18) | -0.68 (-0.72 ‒ -0.64) |
| Bosnia and Herzegovina | 213.59 (139.97 ‒ 324.14) | 191.96 (123.53 ‒ 288.49) | -0.45 (-0.51 ‒ -0.40) |
| Botswana | 578.29 (393.23 ‒ 814.65) | 444.44 (300.92 ‒ 656.59) | -0.92 (-0.95 ‒ -0.89) |
| Brazil | 433.44 (304.04 ‒ 597.68) | 366.62 (253.67 ‒ 507.67) | -0.23 (-0.37 ‒ -0.08) |
| Brunei Darussalam | 151.57 (105.69 ‒ 209.00) | 136.55 (94.84 ‒ 190.61) | -0.37 (-0.40 ‒ -0.34) |
| Bulgaria | 193.66 (124.83 ‒ 296.95) | 183.06 (116.50 ‒ 283.64) | -0.18 (-0.19 ‒ -0.17) |
| Burkina Faso | 544.84 (379.51 ‒ 762.46) | 454.53 (312.92 ‒ 637.79) | -0.65 (-0.68 ‒ -0.63) |
| Burundi | 303.06 (204.42 ‒ 453.05) | 260.38 (169.75 ‒ 390.05) | -0.58 (-0.60 ‒ -0.56) |
| Cabo Verde | 405.10 (277.45 ‒ 574.05) | 326.67 (221.15 ‒ 464.68) | -0.78 (-0.80 ‒ -0.75) |
| Cambodia | 737.23 (509.64 ‒ 1012.60) | 506.57 (353.66 ‒ 709.87) | -1.46 (-1.53 ‒ -1.40) |
| Cameroon | 512.13 (352.48 ‒ 715.72) | 392.27 (266.23 ‒ 552.66) | -1.07 (-1.17 ‒ -0.97) |
| Canada | 101.14 (69.41 ‒ 141.76) | 97.91 (66.98 ‒ 137.02) | -0.12 (-0.13 ‒ -0.11) |
| Central African Republic | 437.79 (297.54 ‒ 615.62) | 403.75 (275.04 ‒ 571.43) | -0.30 (-0.32 ‒ -0.28) |
| Chad | 584.88 (406.32 ‒ 809.59) | 493.60 (340.38 ‒ 686.27) | -0.60 (-0.66 ‒ -0.54) |
| Chile | 183.24 (127.04 ‒ 253.78) | 157.85 (107.23 ‒ 221.48) | -0.49 (-0.50 ‒ -0.48) |
| China | 307.81 (208.57 ‒ 443.00) | 289.22 (194.63 ‒ 422.15) | -0.13 (-0.28 ‒ 0.03) |
| Colombia | 408.17 (282.90 ‒ 567.61) | 334.51 (228.12 ‒ 472.27) | -0.68 (-0.71 ‒ -0.65) |
| Comoros | 464.36 (320.34 ‒ 651.00) | 377.86 (257.06 ‒ 539.69) | -0.74 (-0.76 ‒ -0.71) |
| Congo | 373.69 (253.28 ‒ 537.62) | 312.79 (207.98 ‒ 459.05) | -0.67 (-0.7 ‒ -0.64) |
| Cook Islands | 379.16 (261.06 ‒ 526.01) | 307.76 (210.15 ‒ 436.01) | -0.61 (-0.65 ‒ -0.57) |
| Costa Rica | 353.08 (244.24 ‒ 496.19) | 304.40 (205.14 ‒ 434.58) | -0.54 (-0.58 ‒ -0.51) |
| Croatia | 191.46 (121.92 ‒ 292.01) | 181.78 (113.98 ‒ 280.16) | -0.22 (-0.23 ‒ -0.2) |
| Cuba | 354.22 (247.23 ‒ 493.43) | 307.21 (210.26 ‒ 432.26) | -0.49 (-0.51 ‒ -0.48) |
| Cyprus | 142.76 (98.51 ‒ 199.32) | 125.31 (85.47 ‒ 175.20) | -0.46 (-0.49 ‒ -0.43) |
| Czechia | 192.46 (123.63 ‒ 293.54) | 178.64 (112.53 ‒ 275.29) | -0.22 (-0.26 ‒ -0.18) |
| Cote d'Ivoire | 516.24 (357.31 ‒ 716.49) | 435.54 (299.07 ‒ 613.01) | -0.58 (-0.61 ‒ -0.55) |
| Democratic People's Republic of Korea | 196.36 (120.48 ‒ 322.55) | 170.90 (101.51 ‒ 284.32) | -0.51 (-0.54 ‒ -0.48) |
| Democratic Republic of the Congo | 301.39 (201.49 ‒ 445.14) | 278.54 (184.02 ‒ 414.8) | -0.21 (-0.29 ‒ -0.13) |
| Denmark | 133.57 (92.01 ‒ 187.00) | 124.53 (84.75 ‒ 173.84) | -0.22 (-0.24 ‒ -0.20) |
| Djibouti | 455.04 (313.19 ‒ 639.59) | 366.05 (249.44 ‒ 514.73) | -0.83 (-0.89 ‒ -0.77) |
| Dominica | 269.54 (183.74 ‒ 383.86) | 240.31 (162.43 ‒ 343.48) | -0.4 (-0.44 ‒ -0.36) |
| Dominican Republic | 364.88 (253.27 ‒ 508.08) | 301.01 (204.17 ‒ 426.53) | -0.65 (-0.69 ‒ -0.62) |
| Ecuador | 330.91 (229.31 ‒ 461.00) | 278.41 (188.61 ‒ 396.70) | -0.55 (-0.58 ‒ -0.52) |
| Egypt | 522.76 (365.24 ‒ 716.48) | 395.60 (272.97 ‒ 551.10) | -0.87 (-0.91 ‒ -0.84) |
| El Salvador | 488.16 (341.69 ‒ 672.89) | 383.62 (262.48 ‒ 540.57) | -0.85 (-0.92 ‒ -0.79) |
| Equatorial Guinea | 816.04 (570.8 ‒ 1122.71) | 438.61 (305.47 ‒ 611.74) | -2.41 (-2.55 ‒ -2.28) |
| Eritrea | 584.94 (402.58 ‒ 810.52) | 447.54 (303.97 ‒ 629.53) | -0.86 (-0.88 ‒ -0.84) |
| Estonia | 221.45 (140.57 ‒ 345.29) | 200.47 (123.80 ‒ 321.89) | -0.37 (-0.39 ‒ -0.36) |
| Eswatini | 538.92 (361.49 ‒ 767.14) | 454.53 (304.49 ‒ 676.21) | -0.55 (-0.60 ‒ -0.50) |
| Ethiopia | 730.93 (514.00 ‒ 997.28) | 600.48 (419.44 ‒ 824.26) | -0.68 (-0.70 ‒ -0.66) |
| Fiji | 395.07 (270.98 ‒ 551.89) | 370.75 (256.91 ‒ 524.26) | -0.19 (-0.25 ‒ -0.13) |
| Finland | 136.18 (93.86 ‒ 189.64) | 122.56 (83.63 ‒ 173.06) | -0.37 (-0.40 ‒ -0.34) |
| France | 117.05 (79.29 ‒ 166.55) | 107.66 (71.81 ‒ 154.00) | -0.29 (-0.30 ‒ -0.28) |
| Gabon | 364.28 (247.26 ‒ 523.06) | 297.06 (197.67 ‒ 433.33) | -0.69 (-0.71 ‒ -0.67) |
| Gambia | 481.56 (332.87 ‒ 685.30) | 421.78 (286.86 ‒ 596.37) | -0.31 (-0.39 ‒ -0.24) |
| Georgia | 323.64 (217.22 ‒ 459.19) | 306.1 (205.44 ‒ 443.11) | -0.17 (-0.19 ‒ -0.15) |
| Germany | 133.79 (91.32 ‒ 188.28) | 123.33 (83.84 ‒ 173.43) | -0.26 (-0.28 ‒ -0.24) |
| Ghana | 473.52 (326.28 ‒ 666.36) | 377.61 (254.04 ‒ 542.74) | -0.77 (-0.81 ‒ -0.74) |
| Greece | 125.98 (86.85 ‒ 174.85) | 115.63 (78.97 ‒ 161.66) | -0.28 (-0.31 ‒ -0.25) |
| Greenland | 116.68 (80.58 ‒ 161.56) | 111.08 (76.48 ‒ 154.66) | -0.18 (-0.19 ‒ -0.17) |
| Grenada | 290.98 (198.68 ‒ 409.76) | 242.57 (163.83 ‒ 347.30) | -0.54 (-0.61 ‒ -0.48) |
| Guam | 287.22 (193.82 ‒ 414.54) | 259.39 (174.47 ‒ 376.83) | -0.33 (-0.37 ‒ -0.30) |
| Guatemala | 551.80 (385.74 ‒ 764.27) | 437.93 (300.69 ‒ 609.84) | -0.78 (-0.84 ‒ -0.71) |
| Guinea | 566.82 (393.11 ‒ 789.48) | 472.56 (325.37 ‒ 664.41) | -0.62 (-0.69 ‒ -0.54) |
| Guinea-Bissau | 584.81 (407.78 ‒ 806.87) | 487.41 (334.23 ‒ 686.74) | -0.62 (-0.64 ‒ -0.60) |
| Guyana | 302.18 (208.89 ‒ 425.25) | 257.81 (175.11 ‒ 363.73) | -0.49 (-0.51 ‒ -0.46) |
| Haiti | 364.66 (254.18 ‒ 508.87) | 304.24 (209.47 ‒ 426.70) | -0.62 (-0.64 ‒ -0.59) |
| Honduras | 399.96 (277.81 ‒ 559.32) | 330.70 (224.86 ‒ 470.91) | -0.68 (-0.72 ‒ -0.63) |
| Hungary | 212.32 (139.58 ‒ 316.58) | 191.31 (125.06 ‒ 287.89) | -0.35 (-0.38 ‒ -0.31) |
| Iceland | 143.92 (100.46 ‒ 199.16) | 127.80 (88.51 ‒ 176.68) | -0.41 (-0.45 ‒ -0.37) |
| India | 828.44 (575.31 ‒ 1144.74) | 608.44 (414.87 ‒ 859.67) | -1.10 (-1.13 ‒ -1.06) |
| Indonesia | 819.59 (575.63 ‒ 1113.17) | 644.40 (452.72 ‒ 881.53) | -0.84 (-0.88 ‒ -0.8) |
| Iran (Islamic Republic of) | 568.81 (397.92 ‒ 776.99) | 435.71 (304.53 ‒ 598.99) | -0.90 (-0.98 ‒ -0.81) |
| Iraq | 507.79 (354.77 ‒ 692.36) | 382.54 (265.42 ‒ 530.29) | -1.03 (-1.07 ‒ -1.00) |
| Ireland | 137.36 (94.28 ‒ 191.37) | 123.22 (83.89 ‒ 173.13) | -0.38 (-0.40 ‒ -0.36) |
| Israel | 140.12 (96.62 ‒ 194.67) | 128.28 (87.49 ‒ 179.37) | -0.29 (-0.31 ‒ -0.28) |
| Italy | 243.19 (171.32 ‒ 334.41) | 206.56 (143.31 ‒ 285.41) | -0.59 (-0.66 ‒ -0.51) |
| Jamaica | 272.21 (185.41 ‒ 385.18) | 236.70 (158.57 ‒ 339.49) | -0.49 (-0.53 ‒ -0.45) |
| Japan | 117.65 (80.90 ‒ 163.30) | 110.56 (76.02 ‒ 154.28) | -0.23 (-0.26 ‒ -0.20) |
| Jordan | 369.86 (257.93 ‒ 510.44) | 287.63 (198.08 ‒ 403.27) | -0.94 (-0.98 ‒ -0.91) |
| Kazakhstan | 339.39 (229.77 ‒ 485.7) | 301.49 (202.41 ‒ 438.18) | -0.47 (-0.51 ‒ -0.44) |
| Kenya | 540.23 (377.81 ‒ 744.92) | 429.00 (296.10 ‒ 593.26) | -0.81 (-0.87 ‒ -0.76) |
| Kiribati | 379.43 (264.60 ‒ 530.74) | 341.48 (233.02 ‒ 482.99) | -0.30 (-0.32 ‒ -0.28) |
| Kuwait | 392.82 (272.21 ‒ 545.14) | 323.08 (223.35 ‒ 448.36) | -0.75 (-0.78 ‒ -0.72) |
| Kyrgyzstan | 323.03 (219.98 ‒ 458.71) | 294.30 (198.27 ‒ 423.73) | -0.32 (-0.34 ‒ -0.29) |
| Lao People's Democratic Republic | 357.67 (242.25 ‒ 503.57) | 295.37 (197.68 ‒ 422.71) | -0.67 (-0.69 ‒ -0.64) |
| Latvia | 281.93 (183.41 ‒ 422.35) | 256.29 (165.59 ‒ 390.67) | -0.34 (-0.35 ‒ -0.33) |
| Lebanon | 475.67 (331.87 ‒ 652.36) | 356.55 (246.17 ‒ 495.42) | -1.02 (-1.05 ‒ -0.98) |
| Lesotho | 570.72 (388.35 ‒ 809.94) | 488.19 (327.94 ‒ 708.73) | -0.49 (-0.53 ‒ -0.44) |
| Liberia | 537.74 (374.75 ‒ 748.96) | 442.93 (302.19 ‒ 619.64) | -0.78 (-0.84 ‒ -0.72) |
| Libya | 513.32 (356.82 ‒ 704.46) | 405.72 (281.28 ‒ 562.85) | -0.83 (-0.91 ‒ -0.76) |
| Lithuania | 279.30 (182.94 ‒ 423.13) | 255.35 (165.24 ‒ 387.50) | -0.32 (-0.33 ‒ -0.31) |
| Luxembourg | 133.39 (91.01 ‒ 187.12) | 122.63 (84.33 ‒ 172.04) | -0.28 (-0.30 ‒ -0.26) |
| Madagascar | 381.98 (260.96 ‒ 549.19) | 327.27 (219.35 ‒ 472.39) | -0.55 (-0.58 ‒ -0.52) |
| Malawi | 539.63 (373.21 ‒ 755.71) | 430.29 (295.01 ‒ 606.81) | -0.73 (-0.74 ‒ -0.71) |
| Malaysia | 500.26 (347.70 ‒ 691.93) | 365.15 (251.75 ‒ 510.80) | -1.11 (-1.15 ‒ -1.07) |
| Maldives | 380.37 (259.04 ‒ 535.38) | 297.94 (198.46 ‒ 428.00) | -0.85 (-0.88 ‒ -0.81) |
| Mali | 735.42 (511.49 ‒ 1016.30) | 593.19 (412.00 ‒ 820.79) | -0.68 (-0.70 ‒ -0.65) |
| Malta | 141.33 (97.44 ‒ 196.07) | 127.77 (87.33 ‒ 179.64) | -0.33 (-0.35 ‒ -0.32) |
| Marshall Islands | 366.67 (252.39 ‒ 514.78) | 312.38 (210.86 ‒ 444.13) | -0.55 (-0.57 ‒ -0.53) |
| Mauritania | 509.08 (349.24 ‒ 705.81) | 402.15 (274.23 ‒ 570.01) | -0.79 (-0.81 ‒ -0.77) |
| Mauritius | 422.76 (292.04 ‒ 589.32) | 355.47 (241.73 ‒ 501.48) | -0.55 (-0.57 ‒ -0.53) |
| Mexico | 405.91 (282.57 ‒ 564.41) | 328.89 (222.44 ‒ 463.92) | -0.70 (-0.76 ‒ -0.64) |
| Micronesia (Federated States of) | 357.50 (244.21 ‒ 502.57) | 303.60 (204.48 ‒ 433.83) | -0.55 (-0.56 ‒ -0.53) |
| Monaco | 129.16 (87.97 ‒ 182.74) | 121.86 (82.35 ‒ 170.51) | -0.2 (-0.21 ‒ -0.19) |
| Mongolia | 414.34 (287.07 ‒ 574.48) | 354.04 (240.44 ‒ 500.78) | -0.59 (-0.63 ‒ -0.55) |
| Montenegro | 191.17 (122.95 ‒ 292.09) | 184.06 (117.27 ‒ 281.49) | -0.19 (-0.21 ‒ -0.16) |
| Morocco | 399.60 (279.26 ‒ 553.45) | 324.87 (225.12 ‒ 455.19) | -0.65 (-0.69 ‒ -0.61) |
| Mozambique | 540.94 (377.87 ‒ 753.47) | 436.92 (300.35 ‒ 614.22) | -0.74 (-0.76 ‒ -0.71) |
| Myanmar | 637.1 (449.7 ‒ 876.75) | 479.05 (330.85 ‒ 670.6) | -1.13 (-1.24 ‒ -1.02) |
| Namibia | 537.55 (363.90 ‒ 768.57) | 429.67 (286.64 ‒ 648.02) | -0.78 (-0.83 ‒ -0.74) |
| Nauru | 321.37 (217.97 ‒ 455.95) | 286.70 (192.44 ‒ 412.44) | -0.35 (-0.43 ‒ -0.28) |
| Nepal | 679.69 (449.18 ‒ 972.91) | 536.43 (352.88 ‒ 807.56) | -0.79 (-0.82 ‒ -0.76) |
| Netherlands | 112.27 (75.83 ‒ 159.24) | 103.23 (69.29 ‒ 146.22) | -0.27 (-0.29 ‒ -0.26) |
| New Zealand | 136.58 (93.34 ‒ 191.41) | 124.96 (84.87 ‒ 176.54) | -0.30 (-0.31 ‒ -0.28) |
| Nicaragua | 447.69 (308.96 ‒ 615.22) | 357.27 (244.15 ‒ 503.42) | -0.81 (-0.83 ‒ -0.78) |
| Niger | 616.94 (423.39 ‒ 856.44) | 546.54 (373.76 ‒ 770.38) | -0.52 (-0.56 ‒ -0.48) |
| Nigeria | 646.72 (451.19 ‒ 885.28) | 582.21 (404.70 ‒ 812.30) | -0.45 (-0.51 ‒ -0.39) |
| Niue | 311.95 (210.11 ‒ 445.74) | 267.15 (177.67 ‒ 388.23) | -0.55 (-0.56 ‒ -0.53) |
| North Macedonia | 203.63 (132.04 ‒ 306.02) | 189.29 (121.58 ‒ 290.03) | -0.29 (-0.31 ‒ -0.27) |
| Northern Mariana Islands | 281.37 (188.53 ‒ 406.21) | 259.82 (172.27 ‒ 376.73) | -0.20 (-0.23 ‒ -0.16) |
| Norway | 141.89 (96.48 ‒ 199.09) | 135.35 (91.45 ‒ 191.19) | -0.17 (-0.19 ‒ -0.15) |
| Oman | 777.75 (543.01 ‒ 1069.87) | 556.39 (387.62 ‒ 767.60) | -1.12 (-1.18 ‒ -1.05) |
| Pakistan | 716.14 (498.76 ‒ 991.96) | 665.14 (465.06 ‒ 916.02) | -0.43 (-0.55 ‒ -0.31) |
| Palau | 300.97 (203.64 ‒ 432.14) | 265.53 (177.06 ‒ 384.47) | -0.4 (-0.42 ‒ -0.38) |
| Palestine | 486.02 (338.20 ‒ 666.10) | 366.15 (252.12 ‒ 509.38) | -0.85 (-0.92 ‒ -0.78) |
| Panama | 429.27 (300.15 ‒ 593.25) | 360.37 (250.11 ‒ 508.27) | -0.56 (-0.58 ‒ -0.53) |
| Papua New Guinea | 474.88 (326.69 ‒ 659.92) | 421.64 (285.72 ‒ 593.10) | -0.41 (-0.51 ‒ -0.31) |
| Paraguay | 448.43 (310.32 ‒ 624.37) | 359.97 (246.95 ‒ 507.96) | -0.78 (-0.80 ‒ -0.77) |
| Peru | 510.25 (357.35 ‒ 709.67) | 405.22 (278.27 ‒ 566.83) | -0.99 (-1.08 ‒ -0.9) |
| Philippines | 407.40 (282.44 ‒ 564.71) | 364.27 (250.18 ‒ 509.64) | -0.36 (-0.40 ‒ -0.31) |
| Poland | 182.37 (119.93 ‒ 269.68) | 171.59 (112.52 ‒ 256.72) | -0.22 (-0.24 ‒ -0.20) |
| Portugal | 148.46 (102.84 ‒ 206.23) | 130.96 (89.44 ‒ 184.25) | -0.42 (-0.44 ‒ -0.39) |
| Puerto Rico | 244.00 (164.52 ‒ 346.63) | 211.09 (139.79 ‒ 308.5) | -0.53 (-0.56 ‒ -0.51) |
| Qatar | 480.74 (333.57 ‒ 653.68) | 342.50 (237.28 ‒ 478.91) | -1.24 (-1.29 ‒ -1.19) |
| Republic of Korea | 155.26 (106.86 ‒ 214.67) | 125.40 (85.50 ‒ 175.57) | -0.72 (-0.79 ‒ -0.66) |
| Republic of Moldova | 360.31 (240.85 ‒ 525.28) | 341.77 (227.26 ‒ 503.08) | -0.15 (-0.20 ‒ -0.10) |
| Romania | 203.60 (131.17 ‒ 306.52) | 188.18 (122.08 ‒ 286.65) | -0.28 (-0.30 ‒ -0.27) |
| Russian Federation | 305.46 (204.86 ‒ 444.40) | 291.26 (191.32 ‒ 436.96) | -0.29 (-0.35 ‒ -0.24) |
| Rwanda | 337.51 (226.8 ‒ 489.09) | 273.55 (181.13 ‒ 407.20) | -0.88 (-0.95 ‒ -0.82) |
| Saint Kitts and Nevis | 262.96 (178.09 ‒ 373.65) | 225.03 (151.47 ‒ 323.11) | -0.51 (-0.54 ‒ -0.47) |
| Saint Lucia | 282.43 (193.18 ‒ 401.90) | 239.23 (161.11 ‒ 343.24) | -0.55 (-0.59 ‒ -0.50) |
| Saint Vincent and the Grenadines | 286.30 (196.05 ‒ 405.62) | 249.80 (168.20 ‒ 357.28) | -0.46 (-0.49 ‒ -0.43) |
| Samoa | 328.56 (224.05 ‒ 467.13) | 296.09 (198.83 ‒ 421.31) | -0.31 (-0.33 ‒ -0.29) |
| San Marino | 130.97 (89.37 ‒ 184.24) | 122.83 (83.81 ‒ 172.02) | -0.22 (-0.24 ‒ -0.20) |
| Sao Tome and Principe | 500.94 (345.84 ‒ 700.71) | 396.89 (272.3 ‒ 560.41) | -0.84 (-0.88 ‒ -0.81) |
| Saudi Arabia | 911.89 (635.34 ‒ 1256.34) | 541.34 (377.29 ‒ 743.18) | -1.76 (-1.82 ‒ -1.70) |
| Senegal | 546.57 (379.75 ‒ 757.25) | 462.05 (318.17 ‒ 647.68) | -0.56 (-0.63 ‒ -0.50) |
| Serbia | 201.99 (130.8 ‒ 305.61) | 186.71 (121.28 ‒ 284.78) | -0.31 (-0.32 ‒ -0.30) |
| Seychelles | 431.43 (298.30 ‒ 599.89) | 359.09 (244.28 ‒ 500.60) | -0.59 (-0.62 ‒ -0.57) |
| Sierra Leone | 522.69 (363.45 ‒ 731.20) | 443.05 (303.18 ‒ 625.62) | -0.58 (-0.64 ‒ -0.52) |
| Singapore | 160.91 (109.94 ‒ 224.14) | 146.03 (99.77 ‒ 205.15) | -0.35 (-0.37 ‒ -0.33) |
| Slovakia | 196.49 (127.75 ‒ 299.73) | 181.69 (116.79 ‒ 280.37) | -0.25 (-0.27 ‒ -0.23) |
| Slovenia | 188.63 (120.98 ‒ 286.11) | 176.93 (112.81 ‒ 270.53) | -0.24 (-0.27 ‒ -0.22) |
| Solomon Islands | 379.47 (259.58 ‒ 528.3) | 327.71 (220.97 ‒ 468.03) | -0.45 (-0.49 ‒ -0.41) |
| Somalia | 541.22 (375.19 ‒ 759.83) | 491.49 (336.35 ‒ 694.47) | -0.40 (-0.43 ‒ -0.36) |
| South Africa | 465.17 (315.63 ‒ 668.78) | 414.51 (275.87 ‒ 623.44) | -0.47 (-0.54 ‒ -0.40) |
| South Sudan | 857.08 (603.68 ‒ 1179.50) | 726.48 (506.55 ‒ 1003.08) | -0.59 (-0.64 ‒ -0.55) |
| Spain | 271.44 (188.87 ‒ 377.32) | 249.56 (171.54 ‒ 345.66) | -0.23 (-0.26 ‒ -0.19) |
| Sri Lanka | 430.63 (294.31 ‒ 600.00) | 361.09 (243.46 ‒ 516.09) | -0.48 (-0.56 ‒ -0.40) |
| Sudan | 598.42 (417.12 ‒ 825.08) | 438.68 (304.98 ‒ 606.42) | -1.00 (-1.07 ‒ -0.93) |
| Suriname | 346.01 (239.79 ‒ 483.69) | 297.09 (203.43 ‒ 420.59) | -0.55 (-0.57 ‒ -0.52) |
| Sweden | 107.36 (73.96 ‒ 149.28) | 102.32 (70.22 ‒ 143.60) | -0.23 (-0.25 ‒ -0.21) |
| Switzerland | 128.38 (87.53 ‒ 180.18) | 121.02 (82.87 ‒ 170.79) | -0.20 (-0.21 ‒ -0.19) |
| Syrian Arab Republic | 506.83 (356.33 ‒ 689.70) | 382.73 (264.99 ‒ 530.47) | -1.01 (-1.09 ‒ -0.93) |
| Taiwan (Province of China) | 139.91 (81.13 ‒ 236.76) | 124.51 (70.91 ‒ 215.94) | -0.41 (-0.42 ‒ -0.40) |
| Tajikistan | 374.38 (256.44 ‒ 525.35) | 342.29 (231.71 ‒ 492.36) | -0.31 (-0.34 ‒ -0.28) |
| Thailand | 426.13 (288.55 ‒ 601.37) | 319.64 (213.86 ‒ 468.95) | -1.12 (-1.18 ‒ -1.06) |
| Timor-Leste | 709.00 (497.66 ‒ 966.17) | 569.74 (397.81 ‒ 785.59) | -0.92 (-1.09 ‒ -0.75) |
| Togo | 509.99 (351.14 ‒ 712.84) | 432.40 (294.52 ‒ 608.29) | -0.57 (-0.61 ‒ -0.52) |
| Tokelau | 340.25 (231.43 ‒ 479.97) | 280.77 (188.33 ‒ 406.00) | -0.66 (-0.67 ‒ -0.65) |
| Tonga | 252.95 (168.69 ‒ 367.68) | 220.07 (144.3 ‒ 324.28) | -0.46 (-0.47 ‒ -0.45) |
| Trinidad and Tobago | 267.47 (181.67 ‒ 381.04) | 235.22 (157.69 ‒ 347.21) | -0.59 (-0.64 ‒ -0.53) |
| Tunisia | 458.66 (317.46 ‒ 627.74) | 328.42 (226.87 ‒ 452.31) | -1.19 (-1.25 ‒ -1.12) |
| Turkey | 314.82 (218.00 ‒ 437.33) | 243.53 (166.36 ‒ 342.72) | -0.91 (-0.99 ‒ -0.84) |
| Turkmenistan | 402.01 (270.60 ‒ 579.49) | 337.01 (225.8 ‒ 486.81) | -0.65 (-0.73 ‒ -0.57) |
| Tuvalu | 356.98 (244.44 ‒ 499.80) | 298.15 (201.62 ‒ 427.59) | -0.56 (-0.58 ‒ -0.54) |
| Uganda | 391.18 (268.47 ‒ 561.47) | 317.98 (215.50 ‒ 456.22) | -0.82 (-0.87 ‒ -0.76) |
| Ukraine | 266.74 (177.94 ‒ 388.85) | 251.00 (166.85 ‒ 370.45) | -0.25 (-0.27 ‒ -0.23) |
| United Arab Emirates | 443.93 (310.36 ‒ 607.90) | 340.46 (234.02 ‒ 471.15) | -0.89 (-0.96 ‒ -0.83) |
| United Kingdom | 149.48 (102.13 ‒ 209.35) | 142.94 (97.31 ‒ 201.47) | -0.15 (-0.16 ‒ -0.14) |
| United Republic of Tanzania | 654.32 (443.33 ‒ 925.77) | 545.89 (365.54 ‒ 775.41) | -0.66 (-0.74 ‒ -0.58) |
| United States of America | 107.04 (72.90 ‒ 149.86) | 106.31 (72.50 ‒ 149.18) | -0.02 (-0.06 ‒ 0.02) |
| United States Virgin Islands | 255.74 (174.15 ‒ 362.64) | 219.20 (146.31 ‒ 316.04) | -0.55 (-0.59 ‒ -0.51) |
| Uruguay | 147.51 (99.60 ‒ 206.76) | 138.60 (93.10 ‒ 196.48) | -0.22 (-0.23 ‒ -0.21) |
| Uzbekistan | 360.30 (243.82 ‒ 510.71) | 318.02 (213.51 ‒ 454.95) | -0.45 (-0.46 ‒ -0.43) |
| Vanuatu | 282.20 (191.34 ‒ 409.86) | 256.5 (171.85 ‒ 372.12) | -0.31 (-0.34 ‒ -0.29) |
| Venezuela (Bolivarian Republic of) | 388.39 (269.61 ‒ 540.00) | 325.74 (221.54 ‒ 458.06) | -0.55 (-0.61 ‒ -0.49) |
| Viet Nam | 487.47 (337.23 ‒ 673.00) | 354.88 (243.76 ‒ 504.08) | -1.17 (-1.21 ‒ -1.12) |
| Yemen | 510.46 (356.91 ‒ 697.64) | 399.36 (274.35 ‒ 550.01) | -0.95 (-1.00 ‒ -0.90) |
| Zambia | 433.14 (298.75 ‒ 608.10) | 344.51 (231.64 ‒ 495.69) | -0.85 (-0.92 ‒ -0.78) |
| Zimbabwe | 540.64 (367.55 ‒ 787.06) | 485.56 (328.43 ‒ 718.99) | -0.32 (-0.39 ‒ -0.25) |

YLDs, years lived with disability; ASYR, age-standardized YLD rate; EAPC, estimated annual percentage change; UI, uncertainty interval.
