# Supplementary material for: Global, regional, and national years lived with disability due to blindness and vision loss from 1990 to 2019: Findings from the Global Burden of Disease Study 2019
Source: Front Public Health. 2022 Oct 28;10:1033495. doi: 10.3389/fpubh.2022.1033495 (PMC9650182; doi:10.3389/fpubh.2022.1033495)
Supplement: Supplementary file 9 [file Table_3.docx]

**Supplementary Table 3. Age-standardised rates of years lived with disability due to moderate vision loss, severe vision loss, blindness and presbyopia in 2019 and their temporal trend from 1990 to 2019 at the national level.**

|  | Moderate vision loss（per 10^5^ population） | | |  | Severe vision loss (per 10^5^ population） | | |  | Blindness （per 10^5^ population） | | |  | Presbyopia（per 10^5^ population） | | |
| --- | --- | --- | --- | --- | --- | --- | --- | --- | --- | --- | --- | --- | --- | --- | --- |
|  | ASYRs in 1990 | ASYRs in 2019 | EAPC  (1990-2019，95%UI) |  | ASYRs in 1990 | ASYRs in 2019 | EAPC  (1990-2019，95%UI) |  | ASYRs in 1990 | ASYRs in 2019 | EAPC  (1990-2019，95%UI) |  | ASYRs in 1990 | ASYRs in 2019 | EAPC  (1990-2019，95%UI) |
| Afghanistan | 135.89 | 131.44 | -0.11 (-0.12 ‒ -0.09) |  | 142.98 | 123.72 | -0.49 (-0.55 ‒ -0.43) |  | 356.27 | 280.76 | -0.86 (-1.12 ‒ -0.59) |  | 45.54 | 41.81 | -0.33 (-0.38 ‒ -0.27) |
| Albania | 62.22 | 61.06 | -0.08 (-0.10 ‒ -0.06) |  | 31.37 | 29.53 | -0.28 (-0.31 ‒ -0.26) |  | 46.69 | 33.99 | -1.14 (-1.20 ‒ -1.09) |  | 72.41 | 68.49 | -0.22 (-0.23 ‒ -0.20) |
| Algeria | 125.12 | 118.86 | -0.16 (-0.17 ‒ -0.15) |  | 123.04 | 101.49 | -0.67 (-0.70 ‒ -0.64) |  | 203.71 | 122.10 | -1.71 (-1.75 ‒ -1.67) |  | 39.26 | 35.02 | -0.4 (-0.43 ‒ -0.36) |
| American Samoa | 103.94 | 101.15 | -0.08 (-0.09 ‒ -0.07) |  | 60.60 | 53.45 | -0.42 (-0.45 ‒ -0.4) |  | 90.32 | 68.6 | -0.91 (-0.94 ‒ -0.87) |  | 56.22 | 54.15 | -0.13 (-0.16 ‒ -0.10) |
| Andorra | 58.10 | 57.37 | -0.05 (-0.06 ‒ -0.04) |  | 37.63 | 35.91 | -0.13 (-0.14 ‒ -0.12) |  | 28.19 | 23.35 | -0.63 (-0.72 ‒ -0.55) |  | 5.53 | 5.52 | -0.05 (-0.08 ‒ -0.02) |
| Angola | 90.78 | 88.62 | -0.1 (-0.11 ‒ -0.08) |  | 50.61 | 45.05 | -0.39 (-0.43 ‒ -0.35) |  | 193.53 | 122.59 | -1.58 (-1.68 ‒ -1.48) |  | 93.16 | 84.59 | -0.35 (-0.38 ‒ -0.31) |
| Antigua and Barbuda | 75.39 | 72.96 | -0.1 (-0.11 ‒ -0.1) |  | 47.12 | 42.57 | -0.35 (-0.36 ‒ -0.34) |  | 85.21 | 62.06 | -1.11 (-1.17 ‒ -1.05) |  | 54.11 | 49.73 | -0.26 (-0.27 ‒ -0.24) |
| Argentina | 79.47 | 78.60 | -0.01 (-0.02 ‒ 0.01) |  | 57.20 | 54.05 | -0.14 (-0.17 ‒ -0.11) |  | 48.87 | 34.95 | -1.11 (-1.15 ‒ -1.07) |  | 6.46 | 6.13 | -0.18 (-0.20 ‒ -0.15) |
| Armenia | 102.14 | 99.33 | -0.11 (-0.12 ‒ -0.10) |  | 77.89 | 69.54 | -0.41 (-0.43 ‒ -0.39) |  | 93.19 | 64.93 | -1.38 (-1.44 ‒ -1.33) |  | 71.48 | 67.79 | -0.25 (-0.26 ‒ -0.23) |
| Australia | 55.86 | 56.89 | 0.09 (0.04 ‒ 0.14) |  | 28.60 | 29.60 | 0.18 (0.13 ‒ 0.22) |  | 32.62 | 26.68 | -0.61 (-0.7 ‒ -0.53) |  | 9.91 | 7.22 | -1.11 (-1.4 ‒ -0.81) |
| Austria | 59.16 | 57.91 | -0.08 (-0.09 ‒ -0.08) |  | 39.46 | 36.72 | -0.22 (-0.23 ‒ -0.20) |  | 31.45 | 24.79 | -0.78 (-0.84 ‒ -0.72) |  | 5.93 | 5.69 | -0.17 (-0.19 ‒ -0.16) |
| Azerbaijan | 101.81 | 99.14 | -0.10 (-0.11 ‒ -0.09) |  | 76.29 | 69.67 | -0.32 (-0.35 ‒ -0.29) |  | 99.56 | 74.33 | -1.17 (-1.31 ‒ -1.02) |  | 70.23 | 68.02 | -0.17 (-0.21 ‒ -0.13) |
| Bahamas | 74.58 | 72.56 | -0.09 (-0.09 ‒ -0.09) |  | 45.33 | 41.55 | -0.32 (-0.34 ‒ -0.31) |  | 82.90 | 64.24 | -0.91 (-1.00 ‒ -0.81) |  | 51.01 | 48.27 | -0.25 (-0.27 ‒ -0.23) |
| Bahrain | 122.64 | 116.63 | -0.16 (-0.17 ‒ -0.16) |  | 115.28 | 96.07 | -0.64 (-0.66 ‒ -0.62) |  | 177.74 | 102.57 | -2.02 (-2.09 ‒ -1.95) |  | 36.66 | 33.06 | -0.37 (-0.40 ‒ -0.35) |
| Bangladesh | 146.91 | 146.36 | 0.17 (0.08 ‒ 0.25) |  | 127.4 | 116.68 | 0.01 (-0.12 ‒ 0.13) |  | 240.61 | 137.2 | -1.68 (-1.83 ‒ -1.54) |  | 118.93 | 105.85 | -0.35 (-0.37 ‒ -0.32) |
| Barbados | 37.47 | 36.80 | -0.02 (-0.04 ‒ -0.01) |  | 27.9 | 25.94 | -0.22 (-0.23 ‒ -0.20) |  | 30.37 | 22.94 | -0.90 (-0.97 ‒ -0.84) |  | 52.60 | 49.94 | -0.18 (-0.21 ‒ -0.15) |
| Belarus | 87.99 | 85.38 | -0.08 (-0.09 ‒ -0.07) |  | 58.63 | 52.82 | -0.37 (-0.38 ‒ -0.36) |  | 52.52 | 37.23 | -1.34 (-1.44 ‒ -1.23) |  | 93.51 | 87.68 | -0.19 (-0.21 ‒ -0.18) |
| Belgium | 59.04 | 57.79 | -0.08 (-0.08 ‒ -0.08) |  | 39.50 | 36.66 | -0.22 (-0.23 ‒ -0.21) |  | 32.00 | 24.79 | -0.87 (-0.92 ‒ -0.83) |  | 5.96 | 5.69 | -0.18 (-0.20 ‒ -0.16) |
| Belize | 76.71 | 74.00 | -0.11 (-0.11 ‒ -0.10) |  | 51.53 | 45.35 | -0.42 (-0.45 ‒ -0.40) |  | 110.29 | 78.38 | -1.13 (-1.19 ‒ -1.07) |  | 61.13 | 54.31 | -0.36 (-0.39 ‒ -0.34) |
| Benin | 94.75 | 91.54 | -0.12 (-0.13 ‒ -0.11) |  | 91.32 | 80.41 | -0.40 (-0.45 ‒ -0.35) |  | 251.31 | 174.85 | -1.25 (-1.29 ‒ -1.21) |  | 97.19 | 89.11 | -0.28 (-0.30 ‒ -0.27) |
| Bermuda | 74.66 | 72.47 | -0.09 (-0.1 ‒ -0.09) |  | 45.40 | 41.21 | -0.35 (-0.36 ‒ -0.34) |  | 71.23 | 46.78 | -1.48 (-1.58 ‒ -1.39) |  | 51.28 | 47.91 | -0.28 (-0.30 ‒ -0.25) |
| Bhutan | 90.96 | 78.82 | -0.56 (-0.68 ‒ -0.45) |  | 81.24 | 66.43 | -0.7 (-0.72 ‒ -0.68) |  | 141.90 | 71.64 | -2.51 (-2.58 ‒ -2.44) |  | 121.56 | 108.56 | -0.39 (-0.41 ‒ -0.37) |
| Bolivia (Plurinational State of) | 120.60 | 117.62 | -0.06 (-0.07 ‒ -0.06) |  | 86.19 | 79.98 | -0.24 (-0.25 ‒ -0.22) |  | 198.12 | 123.39 | -1.55 (-1.62 ‒ -1.47) |  | 54.36 | 51.34 | -0.17 (-0.20 ‒ -0.14) |
| Bosnia and Herzegovina | 62.74 | 60.97 | -0.12 (-0.13 ‒ -0.10) |  | 31.51 | 29.03 | -0.36 (-0.39 ‒ -0.32) |  | 46.21 | 34.59 | -1.28 (-1.44 ‒ -1.13) |  | 73.12 | 67.36 | -0.31 (-0.35 ‒ -0.28) |
| Botswana | 67.3 | 65.12 | -0.14 (-0.16 ‒ -0.12) |  | 59.78 | 51.94 | -0.44 (-0.46 ‒ -0.43) |  | 291.19 | 178.88 | -1.69 (-1.77 ‒ -1.62) |  | 160.02 | 148.51 | -0.28 (-0.29 ‒ -0.26) |
| Brazil | 124.45 | 120.19 | 0.41 (0.26 ‒ 0.56) |  | 83.48 | 73.50 | -0.29 (-0.38 ‒ -0.20) |  | 185.19 | 135.66 | -0.7 (-0.96 ‒ -0.44) |  | 40.32 | 37.27 | -0.28 (-0.30 ‒ -0.26) |
| Brunei Darussalam | 55.56 | 54.78 | -0.04 (-0.05 ‒ -0.02) |  | 37.89 | 36.16 | -0.24 (-0.27 ‒ -0.20) |  | 52.00 | 39.81 | -0.89 (-0.96 ‒ -0.83) |  | 6.11 | 5.80 | -0.24 (-0.28 ‒ -0.19) |
| Bulgaria | 61.76 | 60.82 | -0.05 (-0.07 ‒ -0.03) |  | 30.07 | 28.51 | -0.19 (-0.21 ‒ -0.17) |  | 33.15 | 27.58 | -0.55 (-0.63 ‒ -0.48) |  | 68.68 | 66.15 | -0.12 (-0.14 ‒ -0.10) |
| Burkina Faso | 89.84 | 87.02 | -0.11 (-0.14 ‒ -0.08) |  | 104.54 | 93.82 | -0.42 (-0.46 ‒ -0.38) |  | 248.8 | 179.12 | -1.15 (-1.19 ‒ -1.11) |  | 101.65 | 94.57 | -0.28 (-0.31 ‒ -0.26) |
| Burundi | 45.02 | 41.30 | -0.31 (-0.37 ‒ -0.25) |  | 60.04 | 51.95 | -0.51 (-0.53 ‒ -0.50) |  | 90.80 | 64.12 | -1.36 (-1.44 ‒ -1.28) |  | 107.21 | 103.01 | -0.16 (-0.19 ‒ -0.12) |
| Cabo Verde | 69.55 | 68.52 | -0.02 (-0.05 ‒ 0.02) |  | 76.92 | 65.27 | -0.57 (-0.59 ‒ -0.55) |  | 165.75 | 111.27 | -1.44 (-1.5 ‒ -1.39) |  | 92.89 | 81.61 | -0.48 (-0.50 ‒ -0.46) |
| Cambodia | 163.91 | 148.56 | -0.45 (-0.55 ‒ -0.35) |  | 127.55 | 115.44 | -0.43 (-0.57 ‒ -0.29) |  | 377.10 | 180.49 | -2.87 (-2.99 ‒ -2.75) |  | 68.68 | 62.08 | -0.36 (-0.39 ‒ -0.33) |
| Cameroon | 93.83 | 81.83 | -0.73 (-0.88 ‒ -0.58) |  | 98.49 | 83.47 | -0.70 (-0.78 ‒ -0.62) |  | 228.61 | 143.66 | -1.79 (-1.93 ‒ -1.64) |  | 91.20 | 83.31 | -0.3 (-0.33 ‒ -0.27) |
| Canada | 41.03 | 40.69 | -0.04 (-0.04 ‒ -0.03) |  | 30.88 | 29.62 | -0.13 (-0.14 ‒ -0.12) |  | 20.85 | 19.58 | -0.26 (-0.3 ‒ -0.23) |  | 8.37 | 8.02 | -0.15 (-0.16 ‒ -0.14) |
| Central African Republic | 91.19 | 89.93 | -0.07 (-0.08 ‒ -0.05) |  | 51.51 | 48.63 | -0.19 (-0.21 ‒ -0.16) |  | 199.12 | 172.92 | -0.53 (-0.57 ‒ -0.50) |  | 95.98 | 92.27 | -0.12 (-0.14 ‒ -0.09) |
| Chad | 95.73 | 92.10 | -0.15 (-0.16 ‒ -0.14) |  | 106.47 | 92.68 | -0.49 (-0.52 ‒ -0.46) |  | 279.76 | 214.28 | -0.94 (-1.05 ‒ -0.83) |  | 102.93 | 94.54 | -0.29 (-0.32 ‒ -0.26) |
| Chile | 79.19 | 77.23 | -0.07 (-0.09 ‒ -0.06) |  | 39.39 | 35.68 | -0.32 (-0.34 ‒ -0.31) |  | 58.26 | 38.99 | -1.33 (-1.37 ‒ -1.29) |  | 6.41 | 5.95 | -0.31 (-0.34 ‒ -0.28) |
| China | 69.34 | 78.06 | 0.44 (0.31 ‒ 0.56) |  | 43.11 | 44.99 | 0.69 (0.35 ‒ 1.04) |  | 116.38 | 88.52 | -0.8 (-1.01 ‒ -0.58) |  | 78.98 | 77.64 | -0.25 (-0.33 ‒ -0.17) |
| Colombia | 111.77 | 108.33 | -0.11 (-0.11 ‒ -0.11) |  | 82.09 | 73.39 | -0.41 (-0.43 ‒ -0.40) |  | 153.86 | 93.45 | -1.65 (-1.71 ‒ -1.58) |  | 60.45 | 59.34 | -0.14 (-0.16 ‒ -0.12) |
| Comoros | 77.74 | 74.95 | -0.13 (-0.14 ‒ -0.12) |  | 90.77 | 78.66 | -0.47 (-0.49 ‒ -0.44) |  | 193.81 | 129.55 | -1.46 (-1.52 ‒ -1.40) |  | 102.04 | 94.70 | -0.26 (-0.27 ‒ -0.24) |
| Congo | 105.86 | 102.78 | -0.13 (-0.15 ‒ -0.11) |  | 51.73 | 46.29 | -0.38 (-0.42 ‒ -0.35) |  | 129.37 | 85.06 | -1.56 (-1.65 ‒ -1.48) |  | 86.73 | 78.65 | -0.36 (-0.38 ‒ -0.33) |
| Cook Islands | 103.13 | 100.58 | -0.07 (-0.08 ‒ -0.07) |  | 59.21 | 51.89 | -0.45 (-0.47 ‒ -0.43) |  | 161.78 | 103.23 | -1.29 (-1.38 ‒ -1.2) |  | 55.03 | 52.05 | -0.20 (-0.21 ‒ -0.18) |
| Costa Rica | 105.10 | 102.29 | -0.09 (-0.10 ‒ -0.08) |  | 60.30 | 55.10 | -0.31 (-0.34 ‒ -0.28) |  | 128.90 | 89.17 | -1.34 (-1.41 ‒ -1.28) |  | 58.78 | 57.85 | -0.11 (-0.13 ‒ -0.08) |
| Croatia | 61.93 | 60.68 | -0.08 (-0.09 ‒ -0.06) |  | 29.52 | 28.27 | -0.19 (-0.22 ‒ -0.17) |  | 32.71 | 27.72 | -0.67 (-0.7 ‒ -0.63) |  | 67.30 | 65.11 | -0.15 (-0.17 ‒ -0.14) |
| Cuba | 94.24 | 91.83 | -0.09 (-0.12 ‒ -0.07) |  | 66.07 | 60.68 | -0.29 (-0.32 ‒ -0.27) |  | 138.65 | 102.74 | -1.04 (-1.09 ‒ -1.00) |  | 55.26 | 51.95 | -0.20 (-0.24 ‒ -0.17) |
| Cyprus | 58.96 | 57.77 | -0.08 (-0.08 ‒ -0.07) |  | 40.35 | 36.79 | -0.30 (-0.32 ‒ -0.27) |  | 37.27 | 25.04 | -1.46 (-1.55 ‒ -1.36) |  | 6.17 | 5.71 | -0.24 (-0.26 ‒ -0.21) |
| Czechia | 61.77 | 60.36 | -0.08 (-0.10 ‒ -0.07) |  | 29.34 | 27.71 | -0.20 (-0.23 ‒ -0.17) |  | 34.14 | 27.21 | -0.64 (-0.74 ‒ -0.54) |  | 67.21 | 63.36 | -0.16 (-0.2 ‒ -0.13) |
| Cote d'Ivoire | 93.51 | 90.50 | -0.11 (-0.12 ‒ -0.11) |  | 98.43 | 85.93 | -0.44 (-0.46 ‒ -0.41) |  | 230.84 | 172.90 | -1.00 (-1.06 ‒ -0.94) |  | 93.46 | 86.21 | -0.26 (-0.28 ‒ -0.24) |
| Democratic People's Republic of Korea | 57.91 | 54.86 | -0.19 (-0.21 ‒ -0.17) |  | 24.40 | 17.27 | -1.27 (-1.34 ‒ -1.20) |  | 25.96 | 15.82 | -1.78 (-1.94 ‒ -1.63) |  | 88.08 | 82.94 | -0.23 (-0.25 ‒ -0.21) |
| Democratic Republic of the Congo | 77.39 | 75.42 | -0.10 (-0.11 ‒ -0.08) |  | 46.70 | 43.33 | -0.21 (-0.28 ‒ -0.13) |  | 85.13 | 71.45 | -0.41 (-0.58 ‒ -0.24) |  | 92.17 | 88.35 | -0.14 (-0.21 ‒ -0.07) |
| Denmark | 56.12 | 54.93 | -0.05 (-0.07 ‒ -0.03) |  | 47.72 | 44.48 | -0.23 (-0.25 ‒ -0.21) |  | 24.03 | 19.57 | -0.66 (-0.73 ‒ -0.59) |  | 5.70 | 5.55 | -0.11 (-0.16 ‒ -0.07) |
| Djibouti | 77.77 | 74.49 | -0.16 (-0.17 ‒ -0.15) |  | 91.38 | 77.98 | -0.56 (-0.58 ‒ -0.54) |  | 183.43 | 120.62 | -1.60 (-1.74 ‒ -1.46) |  | 102.46 | 92.96 | -0.37 (-0.39 ‒ -0.34) |
| Dominica | 75.58 | 72.90 | -0.12 (-0.12 ‒ -0.11) |  | 47.79 | 42.92 | -0.38 (-0.40 ‒ -0.35) |  | 91.70 | 73.30 | -0.77 (-0.87 ‒ -0.66) |  | 54.47 | 51.19 | -0.25 (-0.27 ‒ -0.23) |
| Dominican Republic | 99.75 | 96.46 | -0.11 (-0.12 ‒ -0.10) |  | 66.04 | 59.06 | -0.43 (-0.47 ‒ -0.39) |  | 138.65 | 90.97 | -1.39 (-1.48 ‒ -1.29) |  | 60.44 | 54.52 | -0.41 (-0.45 ‒ -0.36) |
| Ecuador | 98.14 | 95.96 | -0.06 (-0.07 ‒ -0.05) |  | 66.17 | 62.05 | -0.23 (-0.25 ‒ -0.2) |  | 115.29 | 72.12 | -1.51 (-1.62 ‒ -1.4) |  | 51.31 | 48.28 | -0.18 (-0.21 ‒ -0.16) |
| Egypt | 141.01 | 133.26 | -0.18 (-0.19 ‒ -0.16) |  | 139.68 | 111.01 | -0.74 (-0.76 ‒ -0.72) |  | 202.14 | 116.10 | -1.72 (-1.79 ‒ -1.65) |  | 39.94 | 35.23 | -0.37 (-0.4 ‒ -0.34) |
| El Salvador | 128.43 | 125.89 | -0.07 (-0.08 ‒ -0.06) |  | 88.59 | 78.89 | -0.46 (-0.48 ‒ -0.43) |  | 207.85 | 117.81 | -1.96 (-2.1 ‒ -1.82) |  | 63.29 | 61.04 | -0.22 (-0.26 ‒ -0.18) |
| Equatorial Guinea | 91.18 | 86.72 | -0.21 (-0.23 ‒ -0.19) |  | 51.73 | 41.13 | -0.87 (-0.90 ‒ -0.84) |  | 576.11 | 235.19 | -3.46 (-3.64 ‒ -3.28) |  | 97.01 | 75.57 | -0.97 (-1.00 ‒ -0.93) |
| Eritrea | 83.07 | 79.64 | -0.15 (-0.17 ‒ -0.13) |  | 96.58 | 82.28 | -0.50 (-0.53 ‒ -0.48) |  | 298.42 | 189.54 | -1.42 (-1.47 ‒ -1.37) |  | 106.87 | 96.08 | -0.4 (-0.43 ‒ -0.36) |
| Estonia | 58.72 | 56.70 | -0.1 (-0.11 ‒ -0.09) |  | 42.90 | 38.50 | -0.38 (-0.40 ‒ -0.36) |  | 31.24 | 21.47 | -1.57 (-1.66 ‒ -1.48) |  | 88.59 | 83.8 | -0.19 (-0.21 ‒ -0.17) |
| Eswatini | 68.75 | 66.05 | -0.14 (-0.16 ‒ -0.13) |  | 71.59 | 62.33 | -0.46 (-0.46 ‒ -0.45) |  | 236.43 | 172.47 | -0.99 (-1.13 ‒ -0.85) |  | 162.15 | 153.68 | -0.2 (-0.21 ‒ -0.19) |
| Ethiopia | 103.14 | 97.87 | -0.11 (-0.14 ‒ -0.08) |  | 173.33 | 145.44 | -0.52 (-0.56 ‒ -0.49) |  | 375.29 | 284.68 | -1.00 (-1.05 ‒ -0.96) |  | 79.18 | 72.50 | -0.34 (-0.39 ‒ -0.29) |
| Fiji | 113.43 | 117.87 | 0.14 (0.08 ‒ 0.19) |  | 86.43 | 86.93 | 0.01 (-0.10 ‒ 0.12) |  | 136.00 | 110.15 | -0.62 (-0.68 ‒ -0.56) |  | 59.21 | 55.80 | -0.21 (-0.23 ‒ -0.20) |
| Finland | 59.19 | 57.88 | -0.08 (-0.09 ‒ -0.08) |  | 34.07 | 32.03 | -0.2 (-0.22 ‒ -0.18) |  | 37.08 | 26.98 | -1.12 (-1.21 ‒ -1.03) |  | 5.85 | 5.66 | -0.19 (-0.21 ‒ -0.17) |
| France | 57.76 | 56.30 | -0.09 (-0.10 ‒ -0.08) |  | 32.25 | 30.41 | -0.22 (-0.23 ‒ -0.20) |  | 21.11 | 15.30 | -1.1 (-1.16 ‒ -1.04) |  | 5.93 | 5.65 | -0.15 (-0.17 ‒ -0.14) |
| Gabon | 89.30 | 86.60 | -0.12 (-0.13 ‒ -0.10) |  | 46.78 | 41.18 | -0.41 (-0.44 ‒ -0.39) |  | 142.68 | 93.32 | -1.4 (-1.46 ‒ -1.34) |  | 85.51 | 75.96 | -0.43 (-0.45 ‒ -0.41) |
| Gambia | 87.26 | 93.02 | 0.41 (0.27 ‒ 0.55) |  | 68.37 | 70.62 | 0.36 (0.24 ‒ 0.49) |  | 228.73 | 170.36 | -0.85 (-0.94 ‒ -0.76) |  | 97.20 | 87.79 | -0.36 (-0.36 ‒ -0.35) |
| Georgia | 101.35 | 99.29 | -0.07 (-0.08 ‒ -0.06) |  | 74.60 | 69.49 | -0.22 (-0.25 ‒ -0.19) |  | 80.08 | 69.83 | -0.37 (-0.41 ‒ -0.33) |  | 67.62 | 67.48 | -0.04 (-0.07 ‒ -0.01) |
| Germany | 58.81 | 57.65 | -0.08 (-0.09 ‒ -0.07) |  | 38.23 | 35.86 | -0.19 (-0.20 ‒ -0.17) |  | 31.02 | 24.24 | -0.77 (-0.84 ‒ -0.70) |  | 5.73 | 5.58 | -0.11 (-0.13 ‒ -0.09) |
| Ghana | 118.76 | 117.73 | -0.11 (-0.21 ‒ -0.01) |  | 83.54 | 69.65 | -0.66 (-0.67 ‒ -0.65) |  | 185.32 | 113.88 | -1.58 (-1.62 ‒ -1.54) |  | 85.90 | 76.35 | -0.38 (-0.43 ‒ -0.34) |
| Greece | 50.14 | 49.97 | -0.04 (-0.05 ‒ -0.03) |  | 39.12 | 36.14 | -0.20 (-0.24 ‒ -0.16) |  | 30.57 | 23.69 | -0.85 (-0.95 ‒ -0.76) |  | 6.15 | 5.83 | -0.2 (-0.22 ‒ -0.19) |
| Greenland | 41.64 | 41.03 | -0.06 (-0.06 ‒ -0.06) |  | 32.58 | 30.93 | -0.19 (-0.21 ‒ -0.17) |  | 34.36 | 31.29 | -0.33 (-0.37 ‒ -0.29) |  | 8.09 | 7.82 | -0.11 (-0.12 ‒ -0.10) |
| Grenada | 76.49 | 73.35 | -0.12 (-0.13 ‒ -0.12) |  | 50.13 | 44.12 | -0.4 (-0.44 ‒ -0.37) |  | 105.58 | 72.48 | -1.12 (-1.24 ‒ -0.99) |  | 58.79 | 52.63 | -0.33 (-0.38 ‒ -0.28) |
| Guam | 103.04 | 100.13 | -0.09 (-0.10 ‒ -0.09) |  | 58.26 | 51.31 | -0.47 (-0.48 ‒ -0.45) |  | 71.93 | 56.89 | -0.69 (-0.82 ‒ -0.57) |  | 53.99 | 51.05 | -0.23 (-0.25 ‒ -0.21) |
| Guatemala | 138.74 | 135.12 | -0.05 (-0.07 ‒ -0.03) |  | 113.76 | 100.42 | -0.41 (-0.44 ‒ -0.39) |  | 233.24 | 139.93 | -1.74 (-1.90 ‒ -1.58) |  | 66.06 | 62.46 | -0.25 (-0.27 ‒ -0.22) |
| Guinea | 94.85 | 91.73 | -0.12 (-0.13 ‒ -0.12) |  | 102.85 | 90.14 | -0.45 (-0.47 ‒ -0.43) |  | 269.79 | 199.04 | -1.03 (-1.07 ‒ -0.99) |  | 99.33 | 91.64 | -0.26 (-0.27 ‒ -0.25) |
| Guinea-Bissau | 93.43 | 91.49 | -0.06 (-0.07 ‒ -0.05) |  | 126.13 | 112.02 | -0.37 (-0.44 ‒ -0.30) |  | 266.41 | 193.41 | -1.11 (-1.25 ‒ -0.97) |  | 98.83 | 90.49 | -0.29 (-0.3 ‒ -0.28) |
| Guyana | 76.17 | 73.83 | -0.1 (-0.10 ‒ -0.09) |  | 49.87 | 44.81 | -0.36 (-0.37 ‒ -0.35) |  | 117.51 | 85.59 | -0.94 (-1 ‒ -0.88) |  | 58.63 | 53.57 | -0.3 (-0.31 ‒ -0.29) |
| Haiti | 77.66 | 75.40 | -0.09 (-0.10 ‒ -0.08) |  | 53.55 | 48.54 | -0.34 (-0.36 ‒ -0.33) |  | 169.38 | 121.06 | -1.14 (-1.18 ‒ -1.1) |  | 64.06 | 59.25 | -0.27 (-0.28 ‒ -0.27) |
| Honduras | 111.37 | 108.84 | -0.09 (-0.10 ‒ -0.08) |  | 65.32 | 58.45 | -0.41 (-0.44 ‒ -0.37) |  | 157.86 | 99.61 | -1.59 (-1.67 ‒ -1.5) |  | 65.41 | 63.81 | -0.17 (-0.2 ‒ -0.14) |
| Hungary | 62.01 | 60.51 | -0.10 (-0.12 ‒ -0.07) |  | 29.73 | 28.17 | -0.22 (-0.24 ‒ -0.19) |  | 51.79 | 37.56 | -1.08 (-1.18 ‒ -0.99) |  | 68.79 | 65.09 | -0.15 (-0.18 ‒ -0.11) |
| Iceland | 50.35 | 49.47 | -0.06 (-0.07 ‒ -0.05) |  | 33.47 | 31.14 | -0.19 (-0.22 ‒ -0.17) |  | 54.31 | 41.64 | -0.97 (-1.08 ‒ -0.86) |  | 5.80 | 5.55 | -0.14 (-0.17 ‒ -0.12) |
| India | 200.73 | 170.64 | -0.43 (-0.48 ‒ -0.37) |  | 195.94 | 166.82 | -0.49 (-0.57 ‒ -0.42) |  | 309.85 | 153.11 | -2.57 (-2.65 ‒ -2.50) |  | 121.92 | 117.87 | -0.29 (-0.37 ‒ -0.21) |
| Indonesia | 120.96 | 116.57 | -0.08 (-0.11 ‒ -0.05) |  | 188.06 | 163.72 | -0.53 (-0.57 ‒ -0.48) |  | 463.83 | 321.67 | -1.27 (-1.32 ‒ -1.22) |  | 46.73 | 42.43 | -0.33 (-0.34 ‒ -0.31) |
| Iran (Islamic Republic of) | 136.85 | 124.63 | -0.27 (-0.34 ‒ -0.2) |  | 150.8 | 121.64 | -0.76 (-0.84 ‒ -0.68) |  | 250.22 | 163.15 | -1.45 (-1.60 ‒ -1.29) |  | 30.95 | 26.29 | -0.56 (-0.59 ‒ -0.53) |
| Iraq | 125.38 | 118.83 | -0.18 (-0.18 ‒ -0.17) |  | 125.15 | 100.9 | -0.75 (-0.76 ‒ -0.74) |  | 216.74 | 128.00 | -1.94 (-2.04 ‒ -1.84) |  | 40.52 | 34.82 | -0.49 (-0.51 ‒ -0.47) |
| Ireland | 58.94 | 57.62 | -0.09 (-0.09 ‒ -0.08) |  | 39.63 | 36.46 | -0.26 (-0.27 ‒ -0.24) |  | 32.81 | 23.57 | -1.2 (-1.27 ‒ -1.14) |  | 5.98 | 5.58 | -0.22 (-0.24 ‒ -0.21) |
| Israel | 58.72 | 58.02 | -0.04 (-0.05 ‒ -0.04) |  | 39.86 | 37.36 | -0.19 (-0.2 ‒ -0.18) |  | 35.48 | 27.14 | -0.93 (-0.98 ‒ -0.88) |  | 6.06 | 5.77 | -0.16 (-0.17 ‒ -0.14) |
| Italy | 82.39 | 79.96 | -0.11 (-0.12 ‒ -0.1) |  | 56.56 | 51.86 | -0.28 (-0.3 ‒ -0.26) |  | 99.48 | 70.06 | -1.30 (-1.47 ‒ -1.12) |  | 4.76 | 4.68 | -0.09 (-0.1 ‒ -0.07) |
| Jamaica | 75.88 | 73.34 | -0.11 (-0.11 ‒ -0.1) |  | 48.74 | 43.61 | -0.39 (-0.41 ‒ -0.38) |  | 90.57 | 67.87 | -1.02 (-1.13 ‒ -0.92) |  | 57.02 | 51.88 | -0.33 (-0.35 ‒ -0.31) |
| Japan | 45.12 | 44.99 | 0.01 (-0.01 ‒ 0.03) |  | 35.87 | 35.89 | -0.04 (-0.07 ‒ -0.01) |  | 32.10 | 25.17 | -0.87 (-0.96 ‒ -0.78) |  | 4.55 | 4.52 | -0.09 (-0.11 ‒ -0.07) |
| Jordan | 104.58 | 98.94 | -0.18 (-0.20 ‒ -0.16) |  | 102.69 | 85.44 | -0.67 (-0.69 ‒ -0.64) |  | 124.70 | 69.76 | -2.23 (-2.31 ‒ -2.15) |  | 37.89 | 33.5 | -0.45 (-0.48 ‒ -0.41) |
| Kazakhstan | 102.07 | 99.48 | -0.09 (-0.09 ‒ -0.08) |  | 76.31 | 69.15 | -0.32 (-0.32 ‒ -0.31) |  | 90.98 | 65.93 | -1.34 (-1.49 ‒ -1.18) |  | 70.04 | 66.92 | -0.19 (-0.21 ‒ -0.17) |
| Kenya | 78.04 | 76.09 | -0.13 (-0.18 ‒ -0.08) |  | 79.16 | 68.38 | -0.50 (-0.53 ‒ -0.48) |  | 296.89 | 211.61 | -1.25 (-1.33 ‒ -1.17) |  | 86.14 | 72.93 | -0.39 (-0.45 ‒ -0.33) |
| Kiribati | 106.26 | 104.35 | -0.04 (-0.05 ‒ -0.03) |  | 66.56 | 60.74 | -0.28 (-0.30 ‒ -0.26) |  | 142.94 | 113.41 | -0.68 (-0.72 ‒ -0.64) |  | 63.68 | 62.98 | 0.01 (-0.01 ‒ 0.02) |
| Kuwait | 120.64 | 115.6 | -0.15 (-0.16 ‒ -0.14) |  | 107.93 | 91.39 | -0.65 (-0.69 ‒ -0.62) |  | 129.81 | 84.95 | -1.6 (-1.66 ‒ -1.54) |  | 34.43 | 31.13 | -0.46 (-0.5 ‒ -0.41) |
| Kyrgyzstan | 82.06 | 80.06 | -0.08 (-0.09 ‒ -0.07) |  | 66.84 | 62.23 | -0.19 (-0.2 ‒ -0.17) |  | 101.89 | 80.75 | -0.84 (-0.91 ‒ -0.76) |  | 72.24 | 71.26 | -0.03 (-0.05 ‒ -0.01) |
| Lao People's Democratic Republic | 99.67 | 95.07 | -0.16 (-0.17 ‒ -0.15) |  | 81.99 | 74.62 | -0.36 (-0.39 ‒ -0.33) |  | 107.71 | 64.75 | -1.72 (-1.80 ‒ -1.65) |  | 68.29 | 60.93 | -0.42 (-0.44 ‒ -0.39) |
| Latvia | 87.04 | 84.61 | -0.08 (-0.08 ‒ -0.07) |  | 56.30 | 50.99 | -0.34 (-0.36 ‒ -0.32) |  | 49.46 | 36.64 | -1.23 (-1.30 ‒ -1.16) |  | 89.13 | 84.05 | -0.16 (-0.19 ‒ -0.13) |
| Lebanon | 139.22 | 130.78 | -0.23 (-0.24 ‒ -0.22) |  | 101.51 | 90.12 | -0.32 (-0.4 ‒ -0.24) |  | 196.28 | 101.55 | -2.37 (-2.41 ‒ -2.32) |  | 38.66 | 34.11 | -0.48 (-0.49 ‒ -0.46) |
| Lesotho | 69.34 | 66.73 | -0.14 (-0.15 ‒ -0.12) |  | 74.27 | 64.55 | -0.45 (-0.46 ‒ -0.44) |  | 259.31 | 198.54 | -0.83 (-0.93 ‒ -0.72) |  | 167.81 | 158.37 | -0.17 (-0.18 ‒ -0.15) |
| Liberia | 93.73 | 91.13 | -0.12 (-0.13 ‒ -0.1) |  | 99.43 | 88.15 | -0.47 (-0.52 ‒ -0.42) |  | 248.64 | 175.31 | -1.39 (-1.50 ‒ -1.28) |  | 95.94 | 88.34 | -0.35 (-0.41 ‒ -0.29) |
| Libya | 119.88 | 114.83 | -0.12 (-0.13 ‒ -0.1) |  | 125.69 | 106.45 | -0.59 (-0.64 ‒ -0.55) |  | 229.54 | 151.11 | -1.52 (-1.66 ‒ -1.38) |  | 38.21 | 33.33 | -0.49 (-0.56 ‒ -0.42) |
| Lithuania | 86.87 | 84.46 | -0.08 (-0.09 ‒ -0.07) |  | 56.12 | 50.51 | -0.38 (-0.39 ‒ -0.36) |  | 47.80 | 36.74 | -1.01 (-1.06 ‒ -0.97) |  | 88.51 | 83.63 | -0.19 (-0.21 ‒ -0.18) |
| Luxembourg | 58.65 | 57.51 | -0.08 (-0.08 ‒ -0.07) |  | 38.20 | 36.02 | -0.18 (-0.19 ‒ -0.17) |  | 30.84 | 23.59 | -0.92 (-1.00 ‒ -0.84) |  | 5.69 | 5.51 | -0.12 (-0.17 ‒ -0.08) |
| Madagascar | 75.82 | 73.28 | -0.15 (-0.16 ‒ -0.13) |  | 73.81 | 63.71 | -0.50 (-0.52 ‒ -0.49) |  | 129.30 | 93.19 | -1.14 (-1.21 ‒ -1.06) |  | 103.06 | 97.09 | -0.23 (-0.25 ‒ -0.21) |
| Malawi | 91.87 | 89.07 | -0.06 (-0.09 ‒ -0.03) |  | 118.95 | 98.58 | -0.64 (-0.71 ‒ -0.57) |  | 221.66 | 143.74 | -1.36 (-1.44 ‒ -1.29) |  | 107.15 | 98.90 | -0.3 (-0.32 ‒ -0.28) |
| Malaysia | 124.75 | 113.2 | -0.37 (-0.38 ‒ -0.35) |  | 114.18 | 90.05 | -0.78 (-0.82 ‒ -0.74) |  | 202.65 | 109.42 | -2.19 (-2.30 ‒ -2.08) |  | 58.68 | 52.48 | -0.37 (-0.4 ‒ -0.35) |
| Maldives | 115.26 | 111.93 | -0.12 (-0.13 ‒ -0.11) |  | 73.12 | 61.76 | -0.58 (-0.61 ‒ -0.55) |  | 126.46 | 66.77 | -2.22 (-2.29 ‒ -2.14) |  | 65.53 | 57.49 | -0.44 (-0.46 ‒ -0.42) |
| Mali | 91.32 | 88.65 | -0.09 (-0.11 ‒ -0.07) |  | 105.92 | 94.24 | -0.36 (-0.4 ‒ -0.32) |  | 436.38 | 316.30 | -1.01 (-1.06 ‒ -0.97) |  | 101.8 | 94.00 | -0.26 (-0.27 ‒ -0.24) |
| Malta | 59.41 | 58.12 | -0.08 (-0.08 ‒ -0.07) |  | 40.82 | 37.40 | -0.27 (-0.28 ‒ -0.25) |  | 34.90 | 26.41 | -0.94 (-0.99 ‒ -0.89) |  | 6.20 | 5.84 | -0.22 (-0.25 ‒ -0.18) |
| Marshall Islands | 106.11 | 102.45 | -0.12 (-0.12 ‒ -0.11) |  | 66.91 | 57.25 | -0.54 (-0.56 ‒ -0.52) |  | 129.71 | 93.23 | -1.12 (-1.18 ‒ -1.05) |  | 63.94 | 59.45 | -0.26 (-0.28 ‒ -0.24) |
| Mauritania | 93.68 | 90.44 | -0.13 (-0.14 ‒ -0.12) |  | 97.09 | 83.68 | -0.5 (-0.52 ‒ -0.48) |  | 226.64 | 144.88 | -1.49 (-1.55 ‒ -1.44) |  | 91.67 | 83.15 | -0.31 (-0.33 ‒ -0.29) |
| Mauritius | 127.44 | 122.82 | -0.13 (-0.13 ‒ -0.12) |  | 97.28 | 84.09 | -0.48 (-0.51 ‒ -0.45) |  | 138.46 | 94.63 | -1.18 (-1.23 ‒ -1.13) |  | 59.57 | 53.93 | -0.32 (-0.34 ‒ -0.31) |
| Mexico | 103.98 | 96.81 | -0.18 (-0.21 ‒ -0.15) |  | 111.88 | 95.96 | -0.6 (-0.65 ‒ -0.55) |  | 134.28 | 81.08 | -1.65 (-1.81 ‒ -1.50) |  | 55.76 | 55.05 | -0.07 (-0.08 ‒ -0.05) |
| Micronesia (Federated States of) | 105.43 | 102.75 | -0.08 (-0.08 ‒ -0.07) |  | 65.17 | 57.13 | -0.45 (-0.46 ‒ -0.43) |  | 125.15 | 85.41 | -1.28 (-1.31 ‒ -1.25) |  | 61.76 | 58.31 | -0.19 (-0.2 ‒ -0.17) |
| Monaco | 58.35 | 57.34 | -0.07 (-0.08 ‒ -0.06) |  | 37.85 | 35.81 | -0.16 (-0.18 ‒ -0.15) |  | 27.38 | 23.21 | -0.57 (-0.6 ‒ -0.53) |  | 5.58 | 5.50 | -0.12 (-0.16 ‒ -0.08) |
| Mongolia | 101.69 | 99.59 | -0.07 (-0.07 ‒ -0.06) |  | 71.37 | 64.58 | -0.31 (-0.32 ‒ -0.3) |  | 167.82 | 120.26 | -1.29 (-1.4 ‒ -1.19) |  | 73.46 | 69.61 | -0.18 (-0.19 ‒ -0.16) |
| Montenegro | 61.47 | 60.51 | -0.06 (-0.08 ‒ -0.05) |  | 29.16 | 28.21 | -0.18 (-0.21 ‒ -0.15) |  | 33.58 | 30.38 | -0.50 (-0.61 ‒ -0.39) |  | 66.96 | 64.96 | -0.14 (-0.16 ‒ -0.12) |
| Morocco | 106.14 | 106.34 | 0.08 (0.03 ‒ 0.13) |  | 110.72 | 94.14 | -0.53 (-0.56 ‒ -0.51) |  | 140.72 | 86.89 | -1.54 (-1.61 ‒ -1.47) |  | 42.02 | 37.5 | -0.39 (-0.42 ‒ -0.36) |
| Mozambique | 81.65 | 79.46 | -0.1 (-0.11 ‒ -0.08) |  | 109.89 | 97.17 | -0.42 (-0.47 ‒ -0.37) |  | 237.45 | 158.31 | -1.38 (-1.42 ‒ -1.33) |  | 111.95 | 101.97 | -0.34 (-0.36 ‒ -0.32) |
| Myanmar | 137.41 | 132.62 | -0.12 (-0.13 ‒ -0.1) |  | 106.60 | 90.73 | -0.57 (-0.58 ‒ -0.55) |  | 326.76 | 196.15 | -2.01 (-2.23 ‒ -1.78) |  | 66.33 | 59.56 | -0.44 (-0.47 ‒ -0.4) |
| Namibia | 68.60 | 66.13 | -0.13 (-0.14 ‒ -0.12) |  | 70.17 | 61.57 | -0.44 (-0.45 ‒ -0.42) |  | 239.63 | 152.1 | -1.6 (-1.72 ‒ -1.48) |  | 159.15 | 149.88 | -0.19 (-0.2 ‒ -0.17) |
| Nauru | 104.24 | 102.13 | -0.07 (-0.07 ‒ -0.06) |  | 62.2 | 55.46 | -0.39 (-0.43 ‒ -0.34) |  | 95.91 | 72.29 | -0.83 (-1.04 ‒ -0.62) |  | 59.03 | 56.82 | -0.12 (-0.17 ‒ -0.07) |
| Nepal | 136.97 | 132.66 | -0.15 (-0.17 ‒ -0.14) |  | 135.63 | 122.57 | -0.31 (-0.36 ‒ -0.25) |  | 199.08 | 83.57 | -2.68 (-2.83 ‒ -2.53) |  | 208.01 | 197.62 | -0.32 (-0.38 ‒ -0.27) |
| Netherlands | 51.87 | 50.91 | -0.05 (-0.06 ‒ -0.04) |  | 27.79 | 25.92 | -0.19 (-0.21 ‒ -0.18) |  | 26.81 | 20.79 | -0.9 (-0.94 ‒ -0.85) |  | 5.8 | 5.62 | -0.14 (-0.17 ‒ -0.1) |
| New Zealand | 59.34 | 58.24 | -0.06 (-0.07 ‒ -0.06) |  | 32.97 | 30.95 | -0.21 (-0.22 ‒ -0.2) |  | 38.34 | 30.11 | -0.81 (-0.86 ‒ -0.77) |  | 5.93 | 5.66 | -0.19 (-0.2 ‒ -0.17) |
| Nicaragua | 113.45 | 109.50 | -0.12 (-0.12 ‒ -0.11) |  | 87.15 | 76.95 | -0.44 (-0.46 ‒ -0.42) |  | 182.17 | 108.3 | -1.86 (-1.9 ‒ -1.83) |  | 64.92 | 62.53 | -0.15 (-0.16 ‒ -0.14) |
| Niger | 95.43 | 93.45 | -0.08 (-0.08 ‒ -0.07) |  | 106.76 | 96.71 | -0.32 (-0.34 ‒ -0.31) |  | 293.87 | 217.64 | -1.1 (-1.14 ‒ -1.06) |  | 120.89 | 138.74 | 0.12 (-0.03 ‒ 0.27) |
| Nigeria | 114.63 | 116.78 | 0.06 (-0.04 ‒ 0.16) |  | 153.08 | 137.86 | -0.44 (-0.52 ‒ -0.36) |  | 291.87 | 223.16 | -0.9 (-0.97 ‒ -0.84) |  | 87.15 | 104.41 | 0.06 (-0.18 ‒ 0.3) |
| Niue | 104.54 | 101.38 | -0.09 (-0.10 ‒ -0.08) |  | 61.79 | 53.48 | -0.52 (-0.54 ‒ -0.51) |  | 87.82 | 58.92 | -1.42 (-1.46 ‒ -1.37) |  | 57.79 | 53.37 | -0.28 (-0.3 ‒ -0.27) |
| North Macedonia | 61.69 | 60.53 | -0.07 (-0.08 ‒ -0.05) |  | 30.21 | 28.79 | -0.22 (-0.24 ‒ -0.2) |  | 42.71 | 33.67 | -0.89 (-0.94 ‒ -0.84) |  | 69.03 | 66.29 | -0.19 (-0.21 ‒ -0.17) |
| Northern Mariana Islands | 102.38 | 100.57 | -0.04 (-0.05 ‒ -0.03) |  | 57.47 | 52.44 | -0.28 (-0.3 ‒ -0.27) |  | 68.72 | 54.29 | -0.58 (-0.7 ‒ -0.46) |  | 52.81 | 52.52 | 0.02 (0 ‒ 0.04) |
| Norway | 65.62 | 65.19 | -0.05 (-0.07 ‒ -0.04) |  | 47.7 | 44.45 | -0.19 (-0.21 ‒ -0.17) |  | 23.93 | 21.26 | -0.47 (-0.53 ‒ -0.42) |  | 4.63 | 4.45 | -0.2 (-0.22 ‒ -0.18) |
| Oman | 205.89 | 193.16 | -0.23 (-0.25 ‒ -0.21) |  | 247.2 | 187.25 | -1.01 (-1.04 ‒ -0.97) |  | 284.32 | 143.67 | -2.18 (-2.32 ‒ -2.04) |  | 40.35 | 32.32 | -0.74 (-0.82 ‒ -0.67) |
| Pakistan | 165.67 | 156.51 | -0.28 (-0.43 ‒ -0.13) |  | 184.49 | 151.79 | -0.7 (-0.78 ‒ -0.62) |  | 278.57 | 275.49 | -0.4 (-0.58 ‒ -0.22) |  | 87.42 | 81.36 | -0.26 (-0.28 ‒ -0.24) |
| Palau | 103.80 | 101.02 | -0.08 (-0.08 ‒ -0.07) |  | 60.29 | 53.14 | -0.43 (-0.45 ‒ -0.41) |  | 80.25 | 58.36 | -1.01 (-1.06 ‒ -0.96) |  | 56.63 | 53.02 | -0.2 (-0.21 ‒ -0.19) |
| Palestine | 128.07 | 119.83 | -0.16 (-0.2 ‒ -0.13) |  | 131.45 | 102.22 | -0.81 (-0.85 ‒ -0.77) |  | 183.73 | 108.27 | -1.53 (-1.7 ‒ -1.36) |  | 42.76 | 35.83 | -0.6 (-0.62 ‒ -0.57) |
| Panama | 107.73 | 105.27 | -0.08 (-0.09 ‒ -0.07) |  | 92.72 | 84.57 | -0.32 (-0.34 ‒ -0.3) |  | 170.88 | 113.17 | -1.27 (-1.34 ‒ -1.2) |  | 57.95 | 57.36 | -0.09 (-0.11 ‒ -0.06) |
| Papua New Guinea | 147.73 | 144.51 | -0.12 (-0.25 ‒ 0) |  | 120.35 | 107.23 | -0.42 (-0.55 ‒ -0.29) |  | 139.97 | 106.02 | -0.89 (-0.98 ‒ -0.79) |  | 66.82 | 63.88 | -0.15 (-0.18 ‒ -0.12) |
| Paraguay | 135.26 | 134.76 | 0.04 (0.01 ‒ 0.08) |  | 86.79 | 74.9 | -0.55 (-0.58 ‒ -0.53) |  | 173.91 | 101.41 | -1.95 (-2.02 ‒ -1.89) |  | 52.48 | 48.9 | -0.23 (-0.26 ‒ -0.2) |
| Peru | 144.72 | 140.84 | -0.25 (-0.39 ‒ -0.11) |  | 103.4 | 94.4 | -0.47 (-0.6 ‒ -0.34) |  | 210.88 | 120.41 | -2.23 (-2.35 ‒ -2.1) |  | 51.24 | 49.58 | -0.13 (-0.15 ‒ -0.12) |
| Philippines | 125.17 | 121.91 | -0.21 (-0.28 ‒ -0.13) |  | 77.57 | 69.67 | -0.33 (-0.4 ‒ -0.27) |  | 158.24 | 128.88 | -0.57 (-0.63 ‒ -0.51) |  | 46.42 | 43.82 | -0.13 (-0.17 ‒ -0.09) |
| Poland | 64.34 | 62.78 | -0.09 (-0.11 ‒ -0.07) |  | 31.46 | 29.32 | -0.27 (-0.29 ‒ -0.24) |  | 34.06 | 30.38 | -0.45 (-0.51 ‒ -0.39) |  | 52.51 | 49.11 | -0.21 (-0.24 ‒ -0.18) |
| Portugal | 59.81 | 58.57 | -0.08 (-0.08 ‒ -0.07) |  | 41.98 | 38.56 | -0.26 (-0.28 ‒ -0.25) |  | 40.29 | 27.8 | -1.25 (-1.34 ‒ -1.16) |  | 6.38 | 6.03 | -0.2 (-0.22 ‒ -0.17) |
| Puerto Rico | 74.77 | 72.52 | -0.10 (-0.1 ‒ -0.09) |  | 45.7 | 41.33 | -0.36 (-0.37 ‒ -0.35) |  | 71.46 | 49.35 | -1.38 (-1.46 ‒ -1.31) |  | 52.07 | 47.9 | -0.32 (-0.34 ‒ -0.31) |
| Qatar | 124.11 | 116.73 | -0.20 (-0.23 ‒ -0.18) |  | 118.05 | 91.91 | -0.88 (-0.91 ‒ -0.85) |  | 202.69 | 102.7 | -2.53 (-2.64 ‒ -2.42) |  | 35.88 | 31.16 | -0.49 (-0.54 ‒ -0.45) |
| Republic of Korea | 55.80 | 54.55 | -0.06 (-0.07 ‒ -0.04) |  | 37.76 | 35.4 | -0.28 (-0.31 ‒ -0.25) |  | 55.62 | 29.86 | -2.12 (-2.27 ‒ -1.96) |  | 6.09 | 5.59 | -0.32 (-0.37 ‒ -0.27) |
| Republic of Moldova | 108.58 | 115.35 | 0.34 (0.26 ‒ 0.42) |  | 76.36 | 72.97 | -0.1 (-0.15 ‒ -0.04) |  | 82.9 | 63.43 | -1 (-1.08 ‒ -0.92) |  | 92.47 | 90.02 | -0.09 (-0.11 ‒ -0.08) |
| Romania | 61.95 | 60.93 | -0.06 (-0.08 ‒ -0.04) |  | 30.13 | 28.57 | -0.21 (-0.23 ‒ -0.19) |  | 42.32 | 32.71 | -0.92 (-0.95 ‒ -0.89) |  | 69.2 | 65.97 | -0.17 (-0.19 ‒ -0.15) |
| Russian Federation | 109.18 | 107.31 | -0.04 (-0.06 ‒ -0.03) |  | 61.43 | 55.85 | -0.35 (-0.37 ‒ -0.33) |  | 62.97 | 45.75 | -1.36 (-1.49 ‒ -1.23) |  | 71.89 | 82.35 | 0.15 (0.02 ‒ 0.28) |
| Rwanda | 59.29 | 56.38 | -0.23 (-0.26 ‒ -0.20) |  | 64.67 | 55.51 | -0.58 (-0.62 ‒ -0.55) |  | 110.12 | 64.86 | -2.16 (-2.33 ‒ -2) |  | 103.43 | 96.81 | -0.3 (-0.34 ‒ -0.27) |
| Saint Kitts and Nevis | 75.38 | 72.82 | -0.11 (-0.11 ‒ -0.10) |  | 47.31 | 42.47 | -0.37 (-0.38 ‒ -0.35) |  | 85.85 | 60.07 | -1.17 (-1.26 ‒ -1.09) |  | 54.41 | 49.68 | -0.28 (-0.29 ‒ -0.26) |
| Saint Lucia | 76.14 | 73.48 | -0.11 (-0.11 ‒ -0.10) |  | 49.13 | 43.98 | -0.37 (-0.4 ‒ -0.35) |  | 99.96 | 69.39 | -1.21 (-1.31 ‒ -1.11) |  | 57.21 | 52.38 | -0.29 (-0.31 ‒ -0.27) |
| Saint Vincent and the Grenadines | 76.38 | 73.65 | -0.11 (-0.12 ‒ -0.11) |  | 49.95 | 44.71 | -0.37 (-0.39 ‒ -0.36) |  | 101.69 | 77.3 | -0.93 (-1 ‒ -0.86) |  | 58.28 | 54.13 | -0.26 (-0.27 ‒ -0.24) |
| Samoa | 104.81 | 102.63 | -0.06 (-0.06 ‒ -0.05) |  | 62.96 | 57.2 | -0.32 (-0.35 ‒ -0.3) |  | 101.47 | 77.91 | -0.79 (-0.85 ‒ -0.73) |  | 59.31 | 58.36 | -0.02 (-0.05 ‒ 0) |
| San Marino | 58.42 | 57.33 | -0.08 (-0.08 ‒ -0.07) |  | 38.25 | 36.18 | -0.17 (-0.18 ‒ -0.15) |  | 28.53 | 23.74 | -0.64 (-0.69 ‒ -0.58) |  | 5.76 | 5.58 | -0.11 (-0.14 ‒ -0.08) |
| Sao Tome and Principe | 93.85 | 90.36 | -0.14 (-0.15 ‒ -0.13) |  | 97.87 | 83.64 | -0.55 (-0.58 ‒ -0.53) |  | 216.98 | 140.27 | -1.56 (-1.64 ‒ -1.48) |  | 92.24 | 82.61 | -0.41 (-0.43 ‒ -0.39) |
| Saudi Arabia | 162.19 | 149.41 | -0.28 (-0.3 ‒ -0.27) |  | 195.55 | 136.74 | -1.28 (-1.36 ‒ -1.2) |  | 512.75 | 223.27 | -2.78 (-2.84 ‒ -2.72) |  | 41.4 | 31.92 | -0.87 (-0.94 ‒ -0.79) |
| Senegal | 87.28 | 83.43 | -0.17 (-0.19 ‒ -0.15) |  | 105.56 | 93.9 | -0.39 (-0.43 ‒ -0.35) |  | 258.63 | 196.49 | -0.91 (-1.03 ‒ -0.78) |  | 95.11 | 88.23 | -0.26 (-0.29 ‒ -0.24) |
| Serbia | 61.79 | 60.6 | -0.07 (-0.08 ‒ -0.06) |  | 30.1 | 28.55 | -0.23 (-0.25 ‒ -0.21) |  | 41.08 | 31.99 | -0.97 (-1.02 ‒ -0.93) |  | 69.03 | 65.57 | -0.21 (-0.23 ‒ -0.19) |
| Seychelles | 126.85 | 122.64 | -0.11 (-0.12 ‒ -0.10) |  | 95.69 | 83.74 | -0.41 (-0.44 ‒ -0.37) |  | 150.24 | 99.03 | -1.38 (-1.42 ‒ -1.33) |  | 58.64 | 53.68 | -0.27 (-0.29 ‒ -0.25) |
| Sierra Leone | 97.51 | 95.68 | -0.09 (-0.09 ‒ -0.08) |  | 116.4 | 103.07 | -0.38 (-0.42 ‒ -0.33) |  | 211.29 | 153.61 | -1.12 (-1.25 ‒ -0.99) |  | 97.5 | 90.7 | -0.26 (-0.28 ‒ -0.23) |
| Singapore | 70.47 | 69.04 | -0.05 (-0.06 ‒ -0.04) |  | 40.66 | 38.08 | -0.28 (-0.31 ‒ -0.26) |  | 43.73 | 33.33 | -0.99 (-1.04 ‒ -0.95) |  | 6.05 | 5.59 | -0.27 (-0.3 ‒ -0.25) |
| Slovakia | 61.99 | 60.61 | -0.08 (-0.09 ‒ -0.06) |  | 29.74 | 27.88 | -0.23 (-0.25 ‒ -0.2) |  | 36.68 | 29.19 | -0.74 (-0.77 ‒ -0.7) |  | 68.08 | 64.01 | -0.17 (-0.2 ‒ -0.14) |
| Slovenia | 61.71 | 60.2 | -0.08 (-0.1 ‒ -0.07) |  | 29.01 | 27.68 | -0.18 (-0.21 ‒ -0.16) |  | 31.8 | 25.77 | -0.83 (-0.88 ‒ -0.79) |  | 66.11 | 63.29 | -0.16 (-0.19 ‒ -0.12) |
| Solomon Islands | 106.47 | 103.95 | -0.07 (-0.07 ‒ -0.06) |  | 69.15 | 61.16 | -0.41 (-0.43 ‒ -0.38) |  | 136.81 | 98.83 | -0.97 (-1.06 ‒ -0.89) |  | 67.04 | 63.77 | -0.17 (-0.2 ‒ -0.15) |
| Somalia | 79.76 | 78.07 | -0.08 (-0.09 ‒ -0.08) |  | 101.24 | 92.92 | -0.29 (-0.3 ‒ -0.28) |  | 244.32 | 206.13 | -0.71 (-0.78 ‒ -0.65) |  | 115.91 | 114.38 | -0.06 (-0.08 ‒ -0.05) |
| South Africa | 62.08 | 61.22 | -0.02 (-0.05 ‒ 0.02) |  | 46.45 | 48.7 | 0.39 (0.24 ‒ 0.54) |  | 207.92 | 146.95 | -1.24 (-1.34 ‒ -1.14) |  | 148.72 | 157.64 | -0.04 (-0.15 ‒ 0.06) |
| South Sudan | 133.88 | 130.9 | -0.09 (-0.11 ‒ -0.07) |  | 148.92 | 131.55 | -0.42 (-0.44 ‒ -0.39) |  | 471.93 | 366.46 | -0.9 (-0.97 ‒ -0.83) |  | 102.35 | 97.58 | -0.2 (-0.22 ‒ -0.17) |
| Spain | 98.3 | 99.84 | 0.12 (0.08 ‒ 0.16) |  | 99.44 | 95.25 | -0.07 (-0.11 ‒ -0.03) |  | 67.52 | 48.63 | -1.11 (-1.2 ‒ -1.03) |  | 6.18 | 5.84 | -0.2 (-0.23 ‒ -0.17) |
| Sri Lanka | 145.41 | 140.37 | 0.04 (-0.05 ‒ 0.12) |  | 115.78 | 99.13 | -0.28 (-0.4 ‒ -0.15) |  | 109.59 | 66.77 | -1.73 (-1.82 ‒ -1.64) |  | 59.85 | 54.82 | -0.28 (-0.29 ‒ -0.26) |
| Sudan | 116.72 | 108.9 | -0.24 (-0.25 ‒ -0.22) |  | 123.56 | 99.6 | -0.74 (-0.76 ‒ -0.71) |  | 314.14 | 192.13 | -1.54 (-1.68 ‒ -1.4) |  | 44 | 38.06 | -0.51 (-0.53 ‒ -0.48) |
| Suriname | 90.34 | 88.1 | -0.06 (-0.07 ‒ -0.04) |  | 49.95 | 45.96 | -0.3 (-0.32 ‒ -0.28) |  | 148.46 | 110.29 | -1.08 (-1.11 ‒ -1.04) |  | 57.26 | 52.73 | -0.31 (-0.35 ‒ -0.28) |
| Sweden | 39.23 | 40.87 | -0.01 (-0.06 ‒ 0.05) |  | 34.61 | 32.71 | -0.2 (-0.22 ‒ -0.19) |  | 28.85 | 24.23 | -0.6 (-0.64 ‒ -0.57) |  | 4.67 | 4.51 | -0.2 (-0.23 ‒ -0.16) |
| Switzerland | 58.34 | 57.37 | -0.07 (-0.08 ‒ -0.06) |  | 37.37 | 35.41 | -0.17 (-0.18 ‒ -0.16) |  | 27.23 | 22.75 | -0.6 (-0.66 ‒ -0.54) |  | 5.44 | 5.49 | -0.05 (-0.08 ‒ -0.01) |
| Syrian Arab Republic | 125.34 | 119.38 | -0.16 (-0.17 ‒ -0.15) |  | 124.9 | 102.67 | -0.7 (-0.74 ‒ -0.66) |  | 215.92 | 125.19 | -1.98 (-2.13 ‒ -1.84) |  | 40.66 | 35.49 | -0.49 (-0.54 ‒ -0.44) |
| Taiwan (Province of China) | 46.92 | 45.27 | -0.13 (-0.16 ‒ -0.11) |  | 12.34 | 8.49 | -1.4 (-1.47 ‒ -1.32) |  | 6.1 | 3.02 | -2.55 (-2.62 ‒ -2.47) |  | 74.55 | 67.73 | -0.31 (-0.33 ‒ -0.29) |
| Tajikistan | 102.87 | 100.23 | -0.09 (-0.09 ‒ -0.08) |  | 81.06 | 74.37 | -0.27 (-0.3 ‒ -0.25) |  | 115.51 | 94.99 | -0.7 (-0.77 ‒ -0.63) |  | 74.95 | 72.7 | -0.1 (-0.13 ‒ -0.07) |
| Thailand | 156.5 | 143.41 | -0.38 (-0.46 ‒ -0.31) |  | 71.81 | 57.2 | -0.86 (-0.9 ‒ -0.82) |  | 138.06 | 64.61 | -2.93 (-3.13 ‒ -2.73) |  | 59.76 | 54.43 | -0.31 (-0.33 ‒ -0.3) |
| Timor-Leste | 144.56 | 140.44 | -0.11 (-0.17 ‒ -0.05) |  | 160.43 | 147.17 | -0.34 (-0.47 ‒ -0.21) |  | 335.71 | 221.82 | -1.75 (-2.03 ‒ -1.47) |  | 68.3 | 60.32 | -0.55 (-0.62 ‒ -0.47) |
| Togo | 94.05 | 91.62 | -0.09 (-0.1 ‒ -0.09) |  | 98.87 | 87.42 | -0.39 (-0.42 ‒ -0.37) |  | 222.99 | 167.03 | -1.02 (-1.11 ‒ -0.94) |  | 94.07 | 86.32 | -0.22 (-0.25 ‒ -0.19) |
| Tokelau | 105.25 | 101.57 | -0.12 (-0.13 ‒ -0.11) |  | 64.05 | 54.63 | -0.56 (-0.58 ‒ -0.53) |  | 109.96 | 69.29 | -1.59 (-1.61 ‒ -1.57) |  | 60.99 | 55.28 | -0.33 (-0.35 ‒ -0.31) |
| Tonga | 78.78 | 76.49 | -0.08 (-0.09 ‒ -0.06) |  | 31.75 | 27.89 | -0.47 (-0.51 ‒ -0.44) |  | 82.31 | 58.76 | -1.11 (-1.13 ‒ -1.08) |  | 60.11 | 56.93 | -0.18 (-0.2 ‒ -0.15) |
| Trinidad and Tobago | 79.22 | 77.5 | -0.07 (-0.08 ‒ -0.07) |  | 45.55 | 39.01 | -0.64 (-0.7 ‒ -0.57) |  | 88.83 | 60.34 | -1.51 (-1.63 ‒ -1.39) |  | 53.87 | 58.38 | -0.08 (-0.21 ‒ 0.06) |
| Tunisia | 95.72 | 92.66 | -0.08 (-0.1 ‒ -0.06) |  | 97.13 | 80.1 | -0.68 (-0.71 ‒ -0.65) |  | 226.71 | 121.03 | -2.24 (-2.35 ‒ -2.13) |  | 39.11 | 34.63 | -0.43 (-0.47 ‒ -0.39) |
| Turkey | 79.62 | 84.72 | 0.27 (0.14 ‒ 0.41) |  | 58.68 | 52.03 | -0.38 (-0.48 ‒ -0.28) |  | 137.99 | 73.29 | -2.31 (-2.42 ‒ -2.2) |  | 38.53 | 33.49 | -0.48 (-0.5 ‒ -0.46) |
| Turkmenistan | 143.22 | 124.14 | -0.51 (-0.63 ‒ -0.39) |  | 112.55 | 91.6 | -0.73 (-0.82 ‒ -0.64) |  | 74.83 | 53.97 | -1.24 (-1.3 ‒ -1.17) |  | 71.41 | 67.29 | -0.24 (-0.27 ‒ -0.2) |
| Tuvalu | 105.92 | 102.5 | -0.1 (-0.1 ‒ -0.09) |  | 65.83 | 56.66 | -0.51 (-0.52 ‒ -0.49) |  | 121.88 | 81.28 | -1.26 (-1.31 ‒ -1.21) |  | 63.35 | 57.71 | -0.27 (-0.29 ‒ -0.25) |
| Uganda | 65.81 | 61.71 | -0.27 (-0.3 ‒ -0.24) |  | 78.4 | 64.85 | -0.71 (-0.75 ‒ -0.67) |  | 137.96 | 93.75 | -1.5 (-1.6 ‒ -1.39) |  | 109 | 97.67 | -0.46 (-0.49 ‒ -0.43) |
| Ukraine | 90.27 | 88.41 | -0.05 (-0.06 ‒ -0.04) |  | 58.96 | 54.8 | -0.26 (-0.28 ‒ -0.24) |  | 51.56 | 42.74 | -0.88 (-0.97 ‒ -0.79) |  | 65.96 | 65.04 | -0.06 (-0.07 ‒ -0.04) |
| United Arab Emirates | 121.08 | 113.82 | -0.21 (-0.23 ‒ -0.2) |  | 110.71 | 88.11 | -0.81 (-0.86 ‒ -0.76) |  | 176.79 | 108.27 | -1.61 (-1.72 ‒ -1.49) |  | 35.35 | 30.27 | -0.56 (-0.61 ‒ -0.52) |
| United Kingdom | 70.27 | 68.67 | -0.08 (-0.08 ‒ -0.07) |  | 44.93 | 42.63 | -0.16 (-0.18 ‒ -0.14) |  | 29.32 | 26.88 | -0.31 (-0.32 ‒ -0.31) |  | 4.95 | 4.76 | -0.12 (-0.15 ‒ -0.09) |
| United Republic of Tanzania | 86.3 | 80.39 | -0.24 (-0.26 ‒ -0.22) |  | 115.83 | 101.9 | -0.39 (-0.44 ‒ -0.35) |  | 268.15 | 192.59 | -1.29 (-1.5 ‒ -1.09) |  | 184.03 | 171.02 | -0.22 (-0.24 ‒ -0.2) |
| United States of America | 43.57 | 44 | 0.06 (0.03 ‒ 0.09) |  | 32.3 | 30.83 | -0.15 (-0.17 ‒ -0.13) |  | 22.81 | 23.08 | 0.01 (-0.17 ‒ 0.18) |  | 8.35 | 8.4 | 0.02 (0.01 ‒ 0.03) |
| United States Virgin Islands | 74.92 | 72.34 | -0.11 (-0.12 ‒ -0.11) |  | 46.07 | 41.03 | -0.42 (-0.44 ‒ -0.4) |  | 82.08 | 58.47 | -1.23 (-1.34 ‒ -1.12) |  | 52.67 | 47.36 | -0.38 (-0.39 ‒ -0.36) |
| Uruguay | 75.3 | 74.08 | -0.05 (-0.06 ‒ -0.04) |  | 35.62 | 33.67 | -0.21 (-0.22 ‒ -0.19) |  | 30.11 | 24.64 | -0.7 (-0.74 ‒ -0.67) |  | 6.48 | 6.21 | -0.17 (-0.18 ‒ -0.15) |
| Uzbekistan | 102.82 | 99.76 | -0.1 (-0.1 ‒ -0.09) |  | 80.03 | 71.48 | -0.38 (-0.38 ‒ -0.37) |  | 103.73 | 77.19 | -1.07 (-1.13 ‒ -1.01) |  | 73.73 | 69.57 | -0.22 (-0.24 ‒ -0.2) |
| Vanuatu | 92.95 | 91.59 | -0.05 (-0.06 ‒ -0.04) |  | 57.36 | 50 | -0.49 (-0.52 ‒ -0.47) |  | 68.05 | 53.76 | -0.71 (-0.78 ‒ -0.64) |  | 63.85 | 61.15 | -0.16 (-0.18 ‒ -0.15) |
| Venezuela (Bolivarian Republic of) | 97.34 | 94.57 | -0.08 (-0.09 ‒ -0.07) |  | 71.77 | 65.62 | -0.31 (-0.32 ‒ -0.29) |  | 159.41 | 105.98 | -1.32 (-1.47 ‒ -1.17) |  | 59.88 | 59.57 | 0.02 (-0.02 ‒ 0.07) |
| Viet Nam | 118.43 | 110.54 | -0.31 (-0.36 ‒ -0.27) |  | 102 | 82.14 | -0.74 (-0.75 ‒ -0.72) |  | 202.26 | 104.9 | -2.35 (-2.45 ‒ -2.26) |  | 64.77 | 57.29 | -0.44 (-0.46 ‒ -0.42) |
| Yemen | 115.01 | 109 | -0.15 (-0.16 ‒ -0.13) |  | 108.71 | 90.52 | -0.71 (-0.75 ‒ -0.66) |  | 240.94 | 160.01 | -1.61 (-1.71 ‒ -1.52) |  | 45.8 | 39.82 | -0.55 (-0.58 ‒ -0.51) |
| Zambia | 78.36 | 75.02 | -0.15 (-0.17 ‒ -0.14) |  | 88.79 | 75.69 | -0.56 (-0.62 ‒ -0.51) |  | 165.11 | 101.6 | -1.78 (-1.94 ‒ -1.62) |  | 100.87 | 92.2 | -0.34 (-0.38 ‒ -0.31) |
| Zimbabwe | 74.36 | 72.78 | -0.06 (-0.08 ‒ -0.04) |  | 121.89 | 104 | -0.56 (-0.63 ‒ -0.49) |  | 181.89 | 148.73 | -0.59 (-0.78 ‒ -0.41) |  | 162.5 | 160.05 | 0.02 (-0.02 ‒ 0.06) |

YLDs, years lived with disability; ASYR, age-standardized YLD rate; EAPC, estimated annual percentage change; UI, uncertainty interval.
